# Toxicological Profile of Ultrapure 2,2′,3,4,4′,5,5′-Heptachlorbiphenyl (PCB 180) in Adult Rats

**DOI:** 10.1371/journal.pone.0104639

**Published:** 2014-08-19

**Authors:** Matti Viluksela, Päivi Heikkinen, Leo T. M. van der Ven, Filip Rendel, Robert Roos, Javier Esteban, Merja Korkalainen, Sanna Lensu, Hanna M. Miettinen, Kari Savolainen, Satu Sankari, Hellmuth Lilienthal, Annika Adamsson, Jorma Toppari, Maria Herlin, Mikko Finnilä, Juha Tuukkanen, Heather A. Leslie, Timo Hamers, Gerd Hamscher, Lauy Al-Anati, Ulla Stenius, Kine-Susann Dervola, Inger-Lise Bogen, Frode Fonnum, Patrik L. Andersson, Dieter Schrenk, Krister Halldin, Helen Håkansson

**Affiliations:** 1 Department of Environmental Health, National Institute for Health and Welfare, Kuopio, Finland; 2 Department of Environmental Science, University of Eastern Finland, Kuopio, Finland; 3 Centre for Health Protection, National Institute for Public Health and the Environment, Bilthoven, The Netherlands; 4 Institute of Environmental Medicine, Karolinska Institutet, Stockholm, Sweden; 5 Instituto de Bioingeniería, Universidad Miguel Hernández de Elche, Elche (Alicante), Spain; 6 Department of Biology of Physical Activity, University of Jyväskylä, Jyväskylä, Finland; 7 ISLAB Laboratory Centre, Kuopio, Finland; 8 Department of Equine and Small Animal Medicine, University of Helsinki, Helsinki, Finland; 9 Center of Toxicology, IPA – Institute for Prevention and Occupational Medicine, German Social Accident Insurance, Ruhr University of Bochum, Bochum, Germany; 10 Department of Physiology, University of Turku, Turku, Finland; 11 Department of Paediatrics, University of Turku, Turku, Finland; 12 Department of Anatomy and Cell Biology, University of Oulu, Oulu, Finland; 13 Institute for Environmental Studies, VU University Amsterdam, Amsterdam, The Netherlands; 14 Institute of Food Chemistry and Food Biotechnology, Justus-Liebig University, Giessen, Germany; 15 Department of Biochemistry, Institute of Basic Medical Sciences, University of Oslo, Oslo, Norway; 16 Chemistry Department, Umeå University, Umeå, Sweden; 17 Food Chemistry and Toxicology, University of Kaiserslautern, Kaiserslautern, Germany; Florida International University, United States of America

## Abstract

PCB 180 is a persistent non-dioxin-like polychlorinated biphenyl (NDL-PCB) abundantly present in food and the environment. Risk characterization of NDL-PCBs is confounded by the presence of highly potent dioxin-like impurities. We used ultrapure PCB 180 to characterize its toxicity profile in a 28-day repeat dose toxicity study in young adult rats extended to cover endocrine and behavioral effects. Using a loading dose/maintenance dose regimen, groups of 5 males and 5 females were given total doses of 0, 3, 10, 30, 100, 300, 1000 or 1700 mg PCB 180/kg body weight by gavage. Dose-responses were analyzed using benchmark dose modeling based on dose and adipose tissue PCB concentrations. Body weight gain was retarded at 1700 mg/kg during loading dosing, but recovered thereafter. The most sensitive endpoint of toxicity that was used for risk characterization was altered open field behavior in females; i.e. increased activity and distance moved in the inner zone of an open field suggesting altered emotional responses to unfamiliar environment and impaired behavioral inhibition. Other dose-dependent changes included decreased serum thyroid hormones with associated histopathological changes, altered tissue retinoid levels, decreased hematocrit and hemoglobin, decreased follicle stimulating hormone and luteinizing hormone levels in males and increased expression of DNA damage markers in liver of females. Dose-dependent hypertrophy of *zona fasciculata* cells was observed in adrenals suggesting activation of cortex. There were gender differences in sensitivity and toxicity profiles were partly different in males and females. PCB 180 adipose tissue concentrations were clearly above the general human population levels, but close to the levels in highly exposed populations. The results demonstrate a distinct toxicological profile of PCB 180 with lack of dioxin-like properties required for assignment of WHO toxic equivalency factor. However, PCB 180 shares several toxicological targets with dioxin-like compounds emphasizing the potential for interactions.

## Introduction

Polychlorinated biphenyls (PCBs) include a number of persistent and potent organic pollutants ubiquitously present in human and animal tissues, food and in the environment. Based on their structure and toxicological properties the group of 209 different PCB congeners is divided into 12 dioxin-like PCB (DL-PCB) congeners and 197 non-dioxin-like PCB (NDL-PCB) congeners. DL-PCBs can adopt a planar conformation, because they have no or only one chlorine substitution in the *ortho* position. They bind to the aryl hydrocarbon receptor (AHR) with high affinity and elicit dioxin-like (DL) toxic effects. In contrast, NDL-PCBs are non-planar, do not bind to AHR and are therefore assumed to have a different toxicological profile that varies depending on chemical structure [1].

NDL-PCBs form the majority of total PCBs in the environment and food, and therefore they form a significant portion of human PCB exposure. A World Health Organization (WHO) mother's milk survey carried out in 2001–2002 on 102 human milk pools from 26 countries world-wide indicated that NDL-PCBs account for 90% of total PCBs [1], [2]. In spite of the abundance of NDL-PCBs their toxicity is poorly characterized in terms of the spectrum of effects and potency. Due to lack of relevant data the Scientific Panel on Contaminants in the Food Chain of the European Food Safety Authority (EFSA) was not able to establish health based guidance values for NDL-PCBs [1].

The main problem with the majority of existing data on NDL-PCB toxicity is the simultaneous presence of highly potent DL congeners that makes it impossible to distinguish the specific effects of NDL-PCBs from those of DL compounds. Even trace levels of DL impurities may have toxicologically significant effects overriding the effects of NDL-PCBs [3]. Typical higher total doses of NDL-PCBs in toxicity studies are on the order of hundreds of mg/kg bw, and even doses below 1 µg WHO-TEQ/kg bw of DL impurities may be of toxicological significance [4]. Thus, DL impurity levels as low as 10 µg WHO-TEQ/g NDL-PCB (0.001%) or even below may confound the outcome. Many previous studies have been carried out using technical PCB mixtures with variable amounts of DL constituents, such as polychlorinated dibenzo-*p*-dioxins and dibenzofurans (PCDD/Fs) or DL-PCBs. In most studies with single NDL-PCB congeners or reconstituted mixtures the levels of DL impurities were not quantified or sufficiently reported. It is therefore likely that the outcome of these studies is variably affected by the simultaneous exposure to DL compounds. Similarly, epidemiological studies have not been able to address specific effects of NDL-PCBs, because humans are always exposed simultaneously to complex mixtures of DL and NDL compounds.

A wide variety of toxic effects, including effects on liver, thyroid function, behavior, central nervous system, endocrine system, reproduction and development and immunology [1], [5], have been ascribed to NDL-PCBs, and the fact that most of them are also characteristic for DL compounds makes it difficult to differentiate between the causative groups of compounds. Overall, for most studied endpoints the potency of NDL-PCBs has been reported to be clearly lower than that of DL-PCB 126, the most potent DL-PCB.

Most toxicity studies on NDL-PCBs have been carried out with 2,2′,4,4′,5,5′-hexachlorobiphenyl (PCB 153). Dietary exposure of rats to PCB 153 (PCDD/F impurities >1.0 µg/g) for 90 days revealed e.g. enlarged fatty livers with cytoplasmic vacuolization, increased activity of liver microsomal xenobiotic metabolizing enzymes, reduced follicle size of the thyroid gland, reduced hepatic and pulmonary vitamin A levels and neurochemical alterations in several regions of brain mainly in females [6]. The no-observable-adverse-effect level (NOAEL) was 0.5 mg/kg diet (equivalent with 34 µg/kg bw/day or a total dose of 3.1 mg/kg bw). Similar but milder alterations were observed in an analogous study with 2,2′,3,3′,4,4′-hexachlorobiphenyl (PCB 128; no PCDD/F impurities detected at detection limit of 1.0 µg/g) [7]. As compared to PCB153, the lower potency of PCB128 was associated with lower tissue concentrations due to faster elimination.

In a National Toxicology Program (NTP) study, PCB 153 (purity >99%, DL impurities not reported) was administered to female rats by oral gavage 5 days per week for up to 2 years [8]. The main toxicological findings included increased liver and kidney weights, increased liver pentoxyresorufin-*O*-deethylase (PROD) and 7-ethoxyresorufin-*O*-deethylase (EROD) activity, hepatocyte hypertrophy, diffuse fatty change and bile duct hyperplasia in the liver, decreased serum thyroid hormone concentrations, follicular cell hypertrophy of the thyroid gland, chronic active inflammation in the ovary and oviduct and inflammation of the uterus. As equivocal evidence for carcinogenic activity 4 cases of cholangioma and one hepatocellular adenoma were observed at high exposure levels.

The present study is focused on improving the risk assessment of NDL-PCBs by providing missing critical health hazard information and clarifying biological mechanisms underlying different toxic effects. As the first step towards understanding the toxicity profile of NDL-PCBs a series of comprehensive *in vitro* screening of 17 different assays and QSAR modeling of 19 ultrapure congeners and several other reference PCBs were carried out [9], [10]. NDL-PCBs were selected using a statistical molecular design covering the entire domain of tri- to hepta-chlorinated NDL-PCBs and including congeners abundant in environmental and human tissue samples [11]. Principal component analysis (PCA) of the data from this screening revealed a multivariate toxicity profile that could be divided into three major clusters: DL-PCBs and two separate NDL-PCB groups. The first NDL-PCB group included smaller, *ortho*-substituted congeners with higher biological activity in most of the assays: PCBs 28, 47, 51, 52, 53, 95, 100, 101, 104 and 136. The second group included the most abundant congeners with high biological activity in three endocrine related assays: PCBs 19, 74, 118, 122, 128, 138, 153, 170, 180 and 190. In order to get insight into the toxicity profile and potency of NDL-PCBs *in vivo* two different types of congeners were selected for 28-day toxicity studies, the heptachlorinated PCB 180 (the present study) and the tetrachlorinated PCB 52 (Roos *et al.*, in preparation). These two congeners were considered of highest priority, because (1) they represent different toxicity profile clusters among NDL-PCBs, (2) both of them are abundant in environmental and human samples belonging to the “six indicator PCBs” [1], and (3) no appropriate toxicity studies were available for either of them.

PCB 180 is a toxicologically significant major indicator PCB, because it is very accumulative due to slow elimination. The estimated elimination half-life is 11.5 years in adult humans [12], 9.8 years in early adolescent children [13] and 90 days in rats [14], [15]. PCB 180 is also able to transfer rapidly across the placental barrier [16]. The specific aims of this study were (1) to establish the toxicological profile of PCB 180 by defining target organs and dose-response relationships (benchmark doses, BMDs) for toxic effects, and (2) to establish the relationship between toxic effects and tissue PCB 180 levels. Hepatic effects observed in the animals of this study were recently reported [17], and effects of *in utero*/lactational exposure to PCB 180 and PCB 52 will be reported separately (Roos *et al.*, in preparation).

## Materials and Methods

### Ethics Statement

All animal work was conducted in strict accordance with relevant national and international guidelines. The study protocol was approved by the National Animal Experiment Board of Finland (license No. ESLH-2006-07965/Ym23).

### Chemicals

PCB 180 (2,2′,3′,4,4′,5,5′-heptachlorobiphenyl; CAS 35065-29-3; molecular weight 395.3; batch No. 6115) was purchased from Chiron, Trondheim, Norway and analysed. In brief, 20 mg PCB 180 was dissolved in n-hexane and applied on an activated carbon column, flushed with n-hexane and then back-flushed with toluene to recover DL contaminants [18]. The toluene fraction was analyzed using a gas chromatograph interfaced with a high resolution mass spectrometer tuned for identification of DL-PCBs and PCDD/Fs. The purity of PCB 180 as stated by the supplier was 98.9% and the analyzed level of dioxin-like impurities as represented by sum of WHO-TEQ contamination was 2.7 ng/g PCB 180. The PCB was dissolved in purity controlled (0.2 pg WHO-TEQ/g) corn oil (Sigma Aldrich, Munich, Germany; batch No. 065K0077) applying the same protocol as described above for PCB 180, which served also as control.

### Animals

Outbred male and female Sprague-Dawley rats (*Rattus norvegicus*) were obtained from Harlan Netherlands (Zeist, The Netherlands). During the study they were kept in a conventional laboratory animal unit subjected regularly to health surveys consisting of serological and bacteriological screening as suggested by FELASA [19]. These surveys indicate that the animals were free of typical rodent pathogens. The rats were acclimatized to the experimental conditions for one week before commencing with dosing. At the start of the treatment the rats were 6 weeks old and the mean body weight (±SD) of males was 186.3±14.1 g and that of females 136.3±6.8 g. Altogether 40 male and 40 female rats were used. Rats were randomized by body weight into 8 experimental groups of 5 males and 5 females. The rats were housed in stainless steel, wire-bottomed cages 5 rats/cage (45×38×19 cm) and given standard pelleted R36 feed (Lactamin, Sweden), and tap water *ad libitum*. The room was artificially illuminated from 7 am to 7 pm, and air-conditioned to provide about 8 air changes per hour. The ambient temperature (mean±SD) was 21.3±0.5°C and the relative humidity 48±7%. The animals were individually identified by a tattoo on pinna, and the treatment groups were labeled with color codes.

### Experimental design

The experimental protocol followed the OECD 407 Guideline on Repeated dose 28-day oral toxicity study in rodents, which was enhanced for detection of endocrine, neurotoxicity, retinoid, bone and DNA damage endpoints. In order to improve the assessment of dose-response relationships [20] the number of rats per gender per dose group was reduced to 5 and the amount of dose groups was increased to 8. To rapidly achieve the kinetic steady state, the total dose was divided into 6 daily loading doses and 3 weekly maintenance doses, which were calculated according to the formula [21].
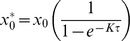
where 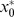  =  loading dose


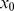
  =  maintenance dose

*K* =  elimination rate constant 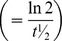



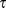
  =  dosing interval

using a half-life 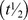 of 90 days [14], [15].

Corn oil (control) or PCB 180 dissolved in corn oil was administered by oral gavage at 4 ml/kg body weight using a metal cannula with a ball tip. Loading doses were administered on days 0–5 and maintenance doses on days 10, 17 and 24 of the study. Selection of the highest dose was based on a pilot study. Experimental groups and doses are given in Table 1.

**Table 1:** Treatment groups and doses. Loading dose was administered on study days 0–5 and maintenance dose on study days 10, 17 and 24.

Treatment group	Total dose (mg/kg bw)	Loading dose (mg/kg bw)	Maintenance dose		Number of animals	
			Weekly dose (mg/kg bw)	Apparent daily dose (µg/kg/day)	Males	Females
1. Control	0	6×0	3×0	0	5	5
2. PCB180	3	6×0.44	3×0.136	19.4	5	5
3. PCB180	10	6×1.44	3×0.45	64.3	5	5
4. PCB180	30	6×4.32	3×1.36	194	5	5
5. PCB180	100	6×14.4	3×4.5	643	5	5
6. PCB180	300	6×43.2	3×14	2000	5	5
7. PCB180	1000	6×144	3×45	6430	5	5
8. PCB180	1700^1^	5^1^×288	3×91	13000	5	5

**Figure 1:**
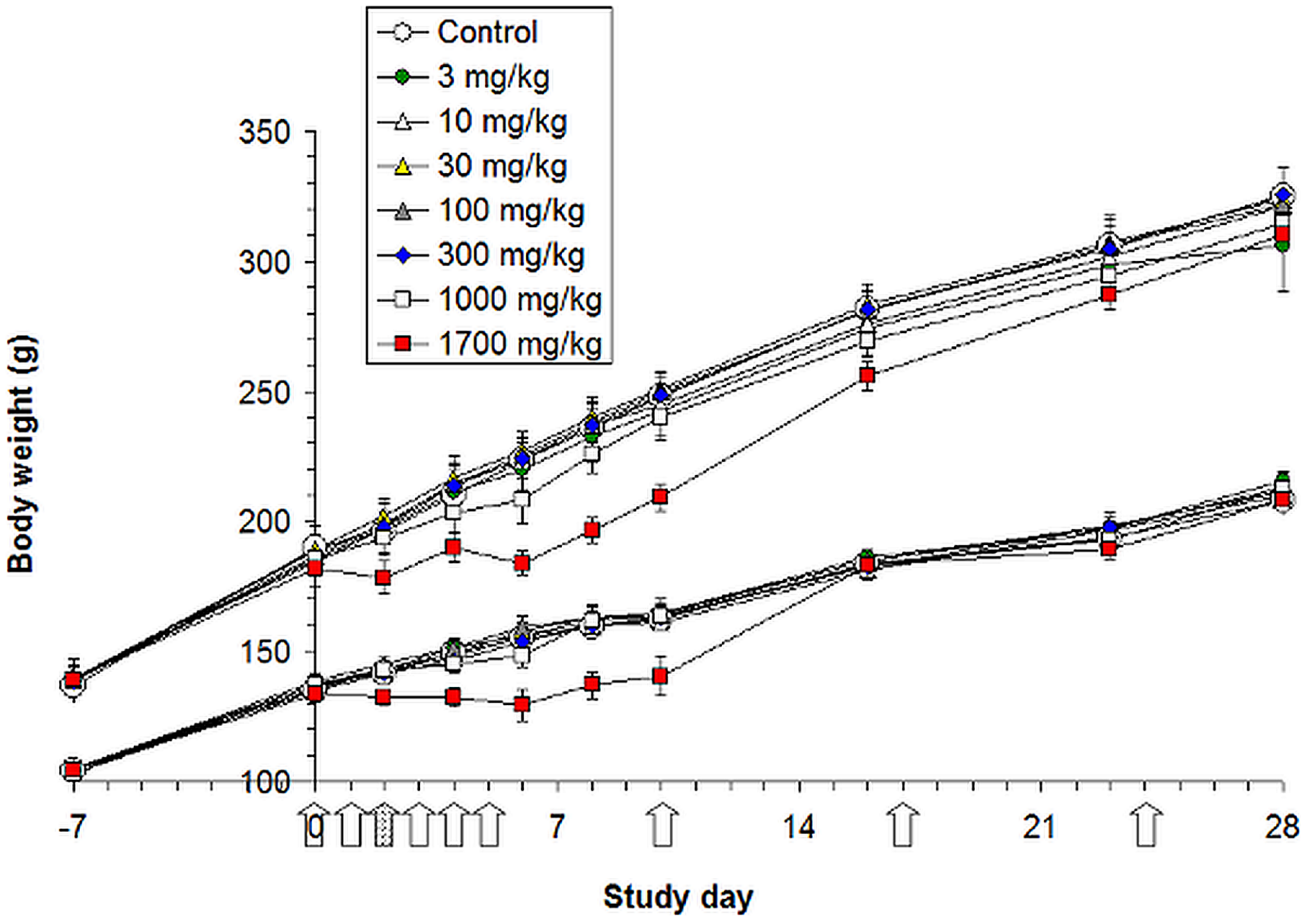
Body weight development of male (upper curves) and female (lower curves) rats. The arrows indicate dosing. Due to unexpected decrease in body weight at the highest dosage group (1700 mg/kg bw) the third loading dose was omitted (dotted arrow) and the rats of this group received only the corn oil vehicle. After loading dose period the body weight development recovered and body weights were similar at the end of the study. Each data point represents mean ± SE (n = 5).

1 The target total dose of group 8 was 2000 mg/kg bw, but due to unexpected decrease in body weight (Fig. 1.) the third loading dose was omitted for animal welfare reasons, and the rats received only the corn oil vehicle.

The rats were observed for clinical signs twice daily except during weekends once daily, and they were weighed every second day during loading dosing period and at least once weekly thereafter. Food and water consumption per cage was recorded once weekly. For determination of the stage of the estrous cycle vaginal smears were collected from female rats daily starting from day 23 of the study. This was done to ensure that the females were at the diestrous stage during necropsy.

At the end of the treatment period (males on study day 28–31, females on study day 28–32) the rats were anesthetized with CO_2_/O_2_ (70/30%). Blood samples were drawn from the left ventricle using Venoject needles (Terumo) and Vacuette EDTA and serum blood collection tubes, and the rats were killed by exsanguination. EDTA blood was used for hematology investigations (see below). Serum was separated, divided into aliquots, frozen in liquid nitrogen and stored at −70°C for further analysis (see below). A complete necropsy (macroscopic observations, tissue sampling for molecular biology, biochemistry, histopathology, analytical chemistry and organ weights) was performed on each rat. The weights of the following organs were recorded: adrenals, brain, epididymides, heart, kidneys, liver, lungs, ovaries, pituitary, prostate (ventral), seminal vesicles, spleen, testes, thymus, thyroids (with parathyroids) and uterus. For molecular biology and biochemical analyses samples from brain, liver, kidney, bones, and several other tissues were snap frozen in liquid nitrogen and stored at −80°C for further analysis. In addition, perirenal adipose tissue and liver samples were stored at −20°C for determination of PCB 180 tissue concentration. Tissue samples for histopathology were preserved in 10% neutral buffered formalin except testis and epididymis, which were fixed in Bouin's solution for 24 h after which they were transferred into 70% ethanol.

### Open field test for locomotor activity

During the last 5 days of the study (days 24–28), rats were tested for locomotor activity in an octagonal open field (diameter 75 cm). The behavior was recorded on videotapes and automatically evaluated with a program for behavioral analyses (Ethovision, Noldus, NL). Each rat was tested for 5 min on each of the 5 days to allow the examination of habituation. Sequence of the rats to be tested was varied according to a permutation scheme to exclude a systematic influence of daytime on the outcome. For analyses, the area of the open field was divided in an inner zone (diameter 50 cm) and an outer ring (width 12.5 cm). Total distance moved during the recording period, distance moved in the inner zone of the open field, distance moved in outer zone, time in inner zone, and time in outer zone were extracted as parameters from the recordings.

### Adipose tissue PCB 180 concentrations

Perirenal adipose tissue samples from each treatment group and gender were pooled (5 individuals per pool), freeze-dried and dry weight was determined. The samples and blanks were extracted by accelerated solvent extraction (ASE) using hexane:dichloromethane (1∶1), followed by an acid silica column cleanup. PCBs 103 and 198 were used as internal standards. Two blanks and a reference material were measured in the series. The extracts were analyzed by gas chromatography with electron capture detection (GC-ECD) with a double column system (CP-SIL 8 CB and CP-SIL 19 CB). Concentrations were calculated using external calibration standards. The concentrations were corrected for the blank signal. Determination of total lipids was performed according to Bligh and Dyer [22].

### Hematology

Basic blood picture analysis was carried out using Advia 120 analyzer (Bayer, later Siemens Diagnostic Division, Dublin, Ireland). This analysis includes red cell count (RBC), hemoglobin (HB), hematocrit (HCT), platelet count (PLT), leukocyte total count (WBC) and differential count as well as the calculated red cell and platelet parameters mean corpuscular volume (MCV), mean corpuscular hemoglobin (MCH), mean corpuscular hemoglobin concentration (MCHC), red cell distribution width - standard deviation (RDW-SD), platelet distribution width (PDW) and mean platelet volume (MPV). Blood cell counting is based on isovolumetric sphering and fixing the cells, stains and light scattering. Visual leukocyte differential counting was done after May-Grünwald-Giemsa staining.

### Clinical chemistry

Spectrophotometric methods were used for the determination of serum total calcium [23], cholesterol [24], creatinine [25], glucose [26], triglycerides [27] and urea [28]. Serum sodium, potassium and chloride concentrations were measured directly by ion selective electrodes. The analyses were performed by a clinical chemistry analyzer (Konelab 30*i*, ThermoFisher Scientific, Vantaa, Finland). Plasma lactate dehydrogenase (LD) activity was measured according to the recommendations of the International Federation of Clinical Chemistry (IFCC 1994/I) using Konelab 60*i* clinical chemistry analyzer. Methods and results for liver related parameters (serum alanine aminotransferase [ALT] activity, alkaline phosphatase [ALP] activity, albumin, total protein and total bilirubin are reported separately [17].

### Thyroid hormones

Serum free triiodothyronine (FT3), free thyroxine (FT4) and thyroid stimulating hormone (TSH) were measured by Elecsys 2010 immunochemistry analyzer using Roche FT3, FT4 and TSH reagents (all from Roche Diagnostics GmbH, Mannheim, Germany). The test principle is electrochemiluminescence immunoassay (ECLIA).

The potencies of 4 theoretically possible mono-hydroxyl metabolites of PCB 180 (3′-OH-PCB 180, 4′-OH-PCB 172, 3′-OH-PCB 182 and 5-OH-PCB 183; structural formulas in Table S13) to compete with T4 for binding to transthyretin (TTR) were determined using a binding assay where the test compound competes with radiolabeled [125I]-T4 for binding to human TTR (Sigma Aldrich) complex during an overnight incubation at 4°C [29]. Full dose-response curves within the range of 1–100 nM were determined for each OH-PCB metabolite.

### Steroids and gonadotropins

Serum testosterone, estradiol and progesterone concentrations were measured by time-resolved immunofluorometric assay, DELFIA (PerkinElmer Life and Analytical Sciences, Turku, Finland) after diethyl ether (Merck KGaA, Darmstadt, Germany) extraction. Ether-extracted serum samples were reconstituted to DELFIA Diluent II buffer (PerkinElmer Life and Analytical Sciences, Turku, Finland) and used for analysis. The sensitivity of the assay was 100 pg/ml for testosterone, 13.6 pg/ml for estradiol and 250 pg/ml for progesterone. The intra- and interassay coefficients of variation (CV) were below 6 and 12%, respectively. To enhance the sensitivity, commercial tracer and antiserum were additionally diluted 5∶8 with assay buffer (PerkinElmer Life and Analytical Sciences, Turku, Finland) in testosterone assay. For estradiol and progesterone, dilution rate was 1∶2. Serum follicle stimulating hormone (FSH) and luteinizing hormone (LH) levels were determined from unextracted samples by DELFIA as described previously [30], [31]. The sensitivity of the rat FSH assay was 0.1 µg/l, intra-assay CV 4.3% and inter-assay CV 10.4% at 4.8 µg/l, and the sensitivity of the rat LH assay was 0.03 µg/l, intra-assay CV 19% at 0.04 µg/l, >5% at >1 µg/l and inter-assay CV 12.5% at 0.24 µg/l and 7.8% at 0.78 µg/l.

### Retinoid analyses

Retinoids in liver, serum, and kidney were analyzed according to Schmidt et al. [32]. The polar retinoids 9-*cis*-retinoic acid (9-*cis*-RA), 13-*cis*-retinoic acid (13-*cis*-RA), 13-*cis*-4-oxo-retinoic acid (13-*cis*-4-o-RA), 9-*cis*-4-oxo-13,14-dihydro-retinoic acid (9-*cis*-4-o-13,14-dh-RA), all-*trans* retinoic acid (all-*trans* RA) and the apolar retinoids retinol and retinyl palmitate were extracted by a single liquid-extraction, separated from each other via solid-phase extraction using an aminopropyl phase, and then injected onto two different HPLC systems that were coupled with UV detection. The polar retinoids were separated on a Spherisorb ODS2 column (2.1×150 mm, 3 µm particle size, Waters, Eschborn, Germany) using a binary gradient and UV detection at 340 nm. The limits of detection (LOD) for all-*trans* RA and 9c-4o-13,14-dh-RA were 0.7 and 1.0 pmol/g tissue, and 0.3 and 0.6 pmol/ml serum, respectively. Retinol and retinyl palmitate were separated on a J'sphere ODS-H80 (4.6×150 mm, 4 µm particle size) obtained from YMC (Schermbeck, Germany), and were detected at 325 nm [32]. The LOD for retinol and retinyl palmitate were 5.6 and 5.5 pmol/g tissue, and 4.2 and 4.2 pmol/ml serum, respectively.

### Liver DNA damage markers

Liver p53, Mdm2 and DNA damage related markers were assessed by Western blotting analysis. Proteins of the liver homogenates were quantified using Coomassie Plus Protein Assay Reagent (Pierce, Täby, Sweden). The samples were subjected to SDS-PAGE, the separated proteins were transferred to a polyvinylidene difluoride membrane (Bio-Rad, Hercules, CA, USA) and blocked in 10% non-fatty milk for 1 h. The protein bands were subsequently probed with antibodies overnight at 4°C.

The primary antibody for total p53 DO-1 was purchased from Novocastra (Newcastle, UK). Primary antibodies for DNA-damage marker were phospho-p53 (Ser-15), phospho-Chk2 (Thr-68), phospho-Mdm2 (Ser-166), phospho-Akt (Ser-473) (Cell Signaling Technology, Stockholm, Sweden) phospho-Erk (Tyr-204) (E-4) and the loading control Cdk2 (M2) (sc-7383 resp. sc-163, Santa Cruz Biotechnology, Santa Cruz, CA, USA). Secondary antibodies used were goat anti-rabbit IgG, goat anti-mouse IgG (sc-2004 resp. sc-2005, Santa Cruz Biotechnology). No signals were obtained when primary antibodies were omitted. Cdk2 was used as a loading control. The results were visualized by the ECL or ECL plus detection kits (Amersham GE Healthcare Bio-sciences AB, Uppsala, Sweden). Cdk2 was used as a loading control. The densitometric analysis was made with Image J version 1.34s software

### Histopathology

After fixation, the samples were dehydrated, paraffinized and embedded according to standard sampling and trimming procedures. Sections of 4 µm were stained with hematoxylin and eosin in an automated way. Microscopic observations were done by initial unblinded comparison of control and top dose samples. Intermediate dose samples were assessed when effects were suggested by initial observations, or otherwise (e.g. effects in organ weights). Slide reading for such affected endpoints was refined by blind and/or semi-quantitative scoring.

For immunohistochemistry of TSH and ACTH in the pituitary routine sections were deparaffinized in a graded series of xylol/ethanol. Endogenous peroxidase activity was blocked in a 1/1 methanol/distilled water solution with 1/100 0.3% H_2_O_2_ added. Antigen exposure was improved by 30 min trypsin incubation (0.25% wt/vol trypsin with 0.02% wt/vol CaCl_2_ in distilled water), and background staining was reduced by 15 min incubation with blocking reagent (Perkin Elmer) and 1% wt/vol in phosphate-buffered solution. Subsequent 60 min incubation with purified rabbit polyclonal IgG against rat TSH (Biogenesis), 1/1600 dilution, or against ACTH (Phoenix Pharmaceuticals), 1/1000 dilution, was followed by incubation with a biotinylated anti-rabbit second antibody in the case of TSH (1/200, 30 min; Vector) and avidin-biotin complex (Vector) according to instructions of the manufacturer; both antisera were diluted in the 1% blocking reagent solution, or with a peroxidase conjugated anti-rabbit second antibody (DAKO) in the case of ACTH. Immunostaining was completed with incubation with a standard diaminobenzidine (Sigma) solution for 5 min and counterstaining with hematoxylin (Mayer procedure). Immunostaining was evaluated by comparing the quantity and staining intensity of positive cells between control and high dose samples.

### Sperm analyses

Frozen right cauda epididymides were homogenized for 2 min using Ultra Turrax homogenizer (model T25 basic, IKA-WERKE GmbH, Staufen, Germany) in 20 ml 0.9% saline containing 0.05% Triton X-100 and 0.01% thiomersal. Homogenates were diluted to about 1×10^6^ sperms/ml, and counts from 4 hemocytometer chambers were counted and averaged.

### Bone geometry, densitometry and biomechanics

Hind limbs were collected and frozen at −20°C. Dissected right tibias were cleaned from soft tissue and stored in Ringer solution at −20°C until analysis. The length of each bone was measured using an electronic sliding caliper to the nearest 0.01 mm (IP65, Sylvac SA, Crissier, Switzerland). The tibias were scanned using the peripheral quantitative computed tomography (pQCT) system (Stratec XCT Research SA+) with software version 5.50 (Norland Stratec Medizintechnik, GmbH, Birkenfeld, Germany) The scans of metaphysis and diaphysis were performed at sites distanced 10% and 45%, respectively, of the length from the growth plate, respectively. The thresholds for defining trabecular bone were 280 and 400 mg/cm^3^, while cortical bone was defined above a threshold of 710 mg/cm^3^. The voxel size was 0.07 mm.

For biomechanical testing hind limbs were defrosted and tibial shafts tested with a three-point bending test using a custom made testing apparatus [33], [34]. Each bone was placed on a support with a span length of 13 mm and bending load was applied with a constant speed of 0.155 mm/sec until failure. The breaking force, bending stiffness and yield force were defined from load-displacement data. Stiffness was calculated as the slope of the linear part of load-displacement curve. Yield force was defined as a corresponding force where the fit for stiffness separated from the measured load-displacement curve. The breaking force was defined as bending load at maximum. Corresponding to these forces also both failure and yield deformations were evaluated.

### Brain amino acid analyses

The right hemisphere of cerebrum was rapidly weighed and homogenized in ice cold 7% HClO_4_ [35] using a glass/teflon Potter-Elvehjem homogenizer (Schwaben Prazision Nordlingen type L43). α-Amino adipic acid was used as internal standard. Samples were neutralized to pH 6.5–7.5 with ice cold KOH/HCl and centrifuged for 20 min at 20 000 g and 4°C in a Sorvall RMC-14-micro centrifuge [36]. Pellets were frozen at −40°C for later protein determination by the BCA-assay [37]. Supernatants were stored at −40°C, filtered with Nylon-66 micro filters (mesh 0.22 µm; Nalgene) prior to HPLC analysis.

Total amino acids in extracts were analyzed, using a reversed-phase HPLC (ChromSpher 5 C18 column; length 25 cm, inner diameter 4.6 mm; Varian) fitted with a fluorescence detector (Shimadzu, Kyoto, Japan), after derivatization with o-phthaldialdehyde (OPA; Sigma) [36]. The mobile phase comprised 75% 50 mM phosphate buffer, pH 5.25, and 25% methanol, changing linearly to 25% phosphate buffer and 75% methanol during 26.5 min after which the methanol concentration was linearly reduced to 15%. Each sample was eluted for 45 min with a flow rate of 0.4 ml/min. A mixture of the amino acids of interest was run as external standards at 100 µM. L-Amino acid standards for aspartic acid, glutamic acid, serine, glutamine, glycine and alanine] were obtained from Pierce (Rockford, Ill., USA), whereas taurine, γ-amino butyric acid, glutathione and α-amino adipic acid standards were from Sigma-Aldrich. Chromatograms were analyzed using the software Lab Solutions (Shimadzu). Both genders of rats exposed to PCB 180 at 0, 10, 30, 300 or 1000 mg/kg bw were analyzed.

### Brain dopamine and nicotinic receptor analyses

Brain homogenate of male rats exposed to PCB 180 at 0 or 1000 mg/kg bw was centrifuged for 30 min at 100 000 g and 4°C using a Beckman Ultracentrifuge. The membrane containing pellet was homogenized in 15 volumes of 50 mM Tris-HCl buffer at pH 7.4, incubated at 25°C for 30 min, and centrifuged again at 100 000 g. The pellet from the second centrifugation was dissolved in 4 ml 0.32 M sucrose/g brain (v/w), with a final protein concentration of 0.25 g/ml and frozen at −40°C.

Dopamine receptors D_1_ and D_5_ were analyzed as described before [38], [39]. In brief, 50 µl (0.7 mg protein) of the membrane preparation was incubated with 124–132 µl buffer (50 mM tris-HCL, 120 mM NaCl, 5 mM KCl, 2,5 mM CaCl_2_ and 1 mM MgCl_2_, pH 7.4) and 100 nM ketanserine (Tocris Bioscience, Bristol, UK; to block binding of SCH23390 to the 5-HT receptor) to a final volume of 200 µl. For measuring unspecific binding 1 nM SCH23390 was added to the same mixture. Finally, 1 nM [^3^H]SCH23390 was added to all samples to measure binding of the receptors or unspecific binding. The samples were incubated for 30 min at 25°C followed by vacuum filtration and scintillation counting.

Nicotinic receptor subunits α4 and β2 were analyzed as above with the following modifications: 8 µl (0.1 mg protein) of the same membrane preparation was incubated with 415–472 µl buffer (50 mM tris-HCL, pH 7.4), 0.1–1 nM [^3^H]epibatidin and 0.1 mM (-)nicotine for measuring unspecific binding, to a final volume of 500 µl. For analysis of low and high affinity binding sites 1 nM and 0.1 nM [^3^H]epibatidin, respectively, were used. The samples were incubated with [^3^H]epibatidin for 1 h followed by vacuum filtration and scintillation counting.

Samples were vacuum filtrated trough Whatman glassfiber-filters (type GF/B, 25 mm) and washed 3×3 ml ice-cold incubation buffer. To reduce unspecific binding, the filters where pre-wetted in 1% polyetylenimine for 60 min. The filters were placed into a filtration bucket and coupled to a mechanical vacuum pump. After filtration the bucket was disassembled and the filters transferred into plastic tubes and 4 ml filtercount fluid was added. Radioactive binding was measured as disintegration per minute (DPM1) in a scintillation counter (Tri-Carb 3100TR, Perkin Elmer). The analyses were carried out in duplicates

### Statistics and margin of exposure calculations

Statistical calculations were performed with the SPSS software package (SPSS Inc., Chicago, IL, USA). For comparisons between groups the equality of variances was first confirmed with Levene's test. If the variances were homogenous one-way analysis of variance (ANOVA) was carried out followed by Tukey HSD test. Retinoid and bone densitometry calculations were performed with Graphpad Prism software version 5.04. For comparisons between groups the equality of variances was first confirmed with Bartlett's test. If the variances were homogenous one-way analysis of variance (ANOVA) was carried out followed by Dunnet's post hoc test. If the variances were heterogeneous even after appropriate transformations, the nonparametric Kruskal-Wallis test was carried out followed by Dunn's test. The limit for statistical significance was p<0.05 (two tailed). ANOVA polynomial and linear contrasts, and the corresponding non-parametric Terpstra-Jonckheere test were used for testing trends. Statistical analyses were conducted separately for males and females.

Data were also analyzed using the BMD method, which is based on dose-response modeling of the full data set using a nested family of exponential and Hill models [20]. The analyses were done on data of males and females combined, using sex as a covariate. The software (PROAST 20.2 and 32.2) then detects significant differences between responses in males and females. In that case, different Critical Effect Doses (CEDs) and CEDs at the lowest bound of the confidence interval (CED-L) were generated for each sex. If no difference between sexes is detected, a CED and CED-Ls for the combined data is generated. However, this was also done if the curve fits are close, and separate analyses for males and females were then performed manually. CEDs and CED-Ls were calculated at the predefined Critical Effect Size (CES) of 5% (EFSA standard) or 10%. Additionally, a CES of 100% was used for CED estimations of intermediate signaling molecules, e.g. DNA damage related protein levels, as they do not directly represent the effect in general.

Margin of exposure (MoE) values were calculated by dividing adipose tissues PCB 180 concentration based CED-L values by human median PCB 180 concentration from different cohorts. They include values from the WHO mother's milk survey in 2001–2002 [1], [2], adipose tissue from the Finnish general population in 1997–1999 [83] and, plasma from the Baltic Sea fisherman cohort in 1997–1999 [41] and placenta from a Danish–Finnish joint prospective cohort in 1997–2001 [40]. WHO has established uncertainty factors (UF) for estimating tolerable intake levels of environmental chemicals [41]. As rat and human tissue concentration data are used no interspecies toxicokinetic UF is needed. A toxicodynamic factor of 2.5 is applied to cover the interspecies variability and a factor of 10 to cover the inter-individual variability in humans. Thus, the UF of 25 is considered adequate for human health endpoints of NDL-PCBs.

### Quality assurance

The in-life phase of the study was carried out according to the principles of Good Laboratory Practice (GLP) at the National Institute for Health and Welfare. The Institute does not have an official GLP status, but the study was audited and site-visited by the internal Quality Assurance Unit.

## Results

Group mean values (±SD) and statistically significant differences from controls for most of the analyzed parameters are presented in Tables S1–S12. Significant dose-responses and the outcome of BMD modeling at CES of 5 and 10% are shown in Tables 2 and 3 based on total dose or adipose tissue PCB 180 concentration, respectively.

**Table 2:** Significant dose-responses of PCB 180 based on total dose.

Parameter			CES 5%			CES 10%			
	Sex	Model	CED (mg/kg bw)	CED-L (mg/kg bw)	Ratio CED/CED-L	CED (mg/kg bw)	CED-L (mg/kg bw)	Ratio CED/CED-L	Maximum response^b^(%)
**Open field behavior**									
Percent time in inner zone, day 24	F	E4	0.35	0.11	3.18	0.71	0.23	3.09	107
Percent distance in inner zone, day 24	F	E4	0.87	0.25	3.48	1.84	0.53	3.47	53.0
Habituation, time	F	E2	184	118	1.56	378	243	1.56	−37.7
Habituation, distance	F	E2	216	138	1.57	444	283	1.57	−33.2
**Hematology**									
Red blood cell count	M	E4	138	82.2	1.67	436	252	1.73	−12.5
Red blood cell count	F	E2	638	514	1.24	1310	1056	1.24	−12.8
Hematocrit	M	E4	140	66.3	2.11	481	NA^a^	NA	−11.8
Hematocrit	F	E2	832	646	1.29	1710	1327	1.29	−10.0
Hemoglobin	M	E4	201	62.8	3.20	NA	NA	NA	−8.60
Hemoglobin	F	E2	739	600	1.23	1520	1233	1.23	−11.1
Platelet count	M	E4	40.7	9.68	4.20	143	NA	NA	12.0
Red cell distribution width - SD	F	E3	1400	1093	1.28	1650	1498	1.10	11.5
**Clinical chemistry**									
Chlolesterol	M	E4	38.3	19.5	1.96	79.0	40.4	1.95	80.8
Chlolesterol	F	E2	192	167	1.15	375	326	1.15	54.1
Triglycerides	M	E4	3.31	1.90	1.74	6.92	3.97	1.74	−62.2
Total protein	M	E4	255	134	1.90	702	379	1.85	14.1
Glucose	M	E2	198	146	1.36	408	300	1.36	−35.6
Glucose	F	E2	677	408	1.66	1390	839	1.66	−12.1
Alkaline phosphatase	M	E2	542	327	1.66	1110	672	1.65	−14.9
Alkaline phosphatase	F	E4	67.6	30.1	2.25	154	68.5	2.25	−25.7
Albumin	M	E2	1550	1090	1.42	3040	2130	1.43	5.50
Potassium	F	E2	1210	786	1.54	2490	1614	1.54	−7.00
Total Bilirubin	F	E4	4.12	0.67	6.13	10.3	1.63	6.32	−17.9
**Thyroid**									
Thyroid gland weight	M	E2	239	168	1.42	467	328	1.42	41.4
Thyroid gland weight	F	E2	443	257	1.72	910	528	1.72	−17.9
Relative thyroid gland weight	M	E2	223	163	1.36	435	319	1.36	45.2
Relative thyroid gland weight	F	E2	468	265	1.76	961	545	1.76	−17.0
Serum free T4 (S-fT4)	M	E4	38.1	26.8	1.42	79.4	56.0	1.42	−66.7
Serum free T4 (S-fT4)	F	E2	128	106.6	1.20	263	219	1.20	−49.4
Serum free T3 (S-fT3)	M	E2	290	218	1.33	596	448	1.33	−26.0
Serum free T3 (S-fT3)	F	E2	354	233	1.52	727	479	1.52	−21.8
Serum TSH^c^	M	E4	0.0747	0.0139	5.37	0.149	0.0278	5.35	5170
Serum TSH^c^	F	E2	32.5	19.9	1.64	63.5	38.8	1.64	1181
Large thyroid follicles (%)	F	E4	22.6	11.6	1.95	131	81.0	1.62	−71.0
**Gonadotropins**									
Serum LH	M	E2	266	150	1.77	547	309	1.77	−27.9
Serum FSH	M	E2	377	263	1.44	775	540	1.44	−20.6
**Retinoids**									
Liver retinol concentration	M	E4	31.9	6.10	5.23	56	10.4	5.40	−46.8
Liver retinol concentration	F	E2	156	119	1.31	374	263	1.42	−42.9
Liver retinyl esters concentration	M	E4	21.5	12.9	1.67	43.9	26.6	1.65	−62.8
Liver retinyl esters concentration	F	E2	122	104	1.17	252	214	1.18	−50.9
Liver all-*trans* RA concentration	M	E2	450	268	1.68	879	523	1.68	20.3
Liver 9*c*-4o-13,14-dh-RA conc.	F	E4	12.2	8.77	1.39	25.3	18.2	1.39	−78.2
Liver retinol amount	M	E2	437	261	1.67	897	536	1.67	−18.1
Liver retinyl esters amount	M	E2	145	123	1.18	296	252	1.17	−45.2
Liver retinyl esters amount	F	E2	254	189	1.34	522	388	1.35	−29.0
Liver all-*trans* RA amount	M	E4	21.7	9.07	2.39	44.7	18.7	2.39	92.7
Liver 9*c*-4o-13,14-dh-RA amount	F	E4	13.1	8.29	1.58	27.2	17.2	1.58	−70.3
Kidney retinol concentration	M	E2	200	149	1.34	390	291	1.34	51.5
Kidney retinol concentration	F	E2	335	216	1.55	655	421	1.56	27.8
Kidney retinyl esters concentration	M	E4	9.88	3.35	2.95	20	6.77	2.95	254
Kidney all-*trans* RA concentration	F	E2	457	321	1.43	892	626	1.42	19.9
Kidney retinol amount	M	E2	206	147	1.40	402	288	1.40	50.4
Kidney retinol amount	F	E2	355	221	1.61	694	431	1.61	26.9
Kidney retinyl esters amount	M	E4	9.14	3.10	2.95	18.5	6.26	2.96	260
Kidney all-*trans* RA amount	F	E2	494	319	1.55	965	625	1.54	16.5
Serum retinol concentration	F	E2	400	242	1.65	781	474	1.65	23.1
**Bone densitometry**									
Cortical area of diaphysis	M	E2	1380	820	1.68	NA	NA	NA	−6.10
Trabecular area of metaphysis	F	E2	1040	602	1.73	NA	NA	NA	8.30
**Bone biomechanics**									
F yield	F	E2	476	266	1.79	978	547	1.79	−16.7
**Organ weights**									
Liver weight	M	E4	11.6	5.48	2.12	24.2	11.4	2.12	66.0
Liver weight	F	E2	225	188	1.19	439	368	1.19	44.6
Relative liver weight	M	E4	15.6	9.84	1.59	32.5	20.5	1.59	67.0
Relative liver weight	F	E2	218	194	1.13	427	378	1.13	46.2
Relative thymus weight	M	E2	527	299	1.76	1030	585	1.76	17.1
Ovaries weight	F	E4	46.8	6.15	7.61	120	15.4	7.78	17.0
**DNA damage markers**						CES 100%			
Liver p53	F	E4	14.1	5.89	2.39	472	210	2.25	142
Liver p53 Ser15	F	E5	79.2	19.5	4.07	101	93.0	1.09	181
Liver pChk2 Thr68	F	E5	72.9	31.5	2.31	81.1	41.8	1.94	185
**Liver enzymes** d									
EROD activity	M		0.40	0.30	1.33	0.70	0.50	1.40	
EROD activity	F		2.20	1.70	1.29	4.40	3.40	1.29	
PROD activity	M		0.90	0.70	1.29	1.30	1.00	1.30	
PROD activity	F		3.50	2.50	1.40	5.10	3.60	1.42	
CYP2B1 mRNA	M		0.70	0.40	1.75	1.00	0.50	2.00	
CYP2B1 mRNA	F		1.70	0.90	1.89	2.20	1.20	1.83	
CYP3A1 mRNA	M		1.40	0.90	1.56	2.90	1.80	1.61	
CYP3A1 mRNA	F		1.50	0.70	2.14	2.90	1.40	2.07	
UGT1A1 mRNA	M		13.3	6.60	2.02	26.9	13.5	1.99	
UGT1A1 mRNA	F		49.0	26.2	1.87	99.0	53.2	1.86	
UGT1A6 mRNA	M		3.20	2.20	1.45	6.50	4.50	1.44	
UGT1A6 mRNA	F		23.6	16.4	1.44	47.4	32.9	1.44	
T4 UGT activity, pooled data m+f	M+F		23.1	13.0	1.78	47.5	26.7	1.78	

a Not available.

b Calculated as the percent difference between controls and high dose according to the fitted model.

c Half min value added to zeros.

d Data from Roos *et al.*, 2011.

**Table 3:** Significant dose-responses of PCB 180 based on adipose tissue concentration.

Parameter			CES 5%			CES 10%			
	Sex	Model	CED (µg/g lipid)	CED-L (µg/g lipid)	Ratio CED/CED-L	CED (µg/g lipid)	CED-L (µg/g lipid)	Ratio CED/CED-L	Maximum response^b^ (%)
**Open field behavior**									
Percent time in inner zone, day 24	F	E4	1.55	0.525	2.95	3.17	1.07	2.96	111
Percent distance in inner zone, day 24	F	E4	4.12	1.16	3.54	8.69	2.423	3.59	53.0
Habituation, time	F	E2	1130	739	1.53	2320	1518	1.53	−40.1
Habituation, distance	F	E2	1340	865	1.55	2740	1777	1.54	−35.2
**Hematology**									
Red blood cell count	M	E4	598	337	1.77	1890	NA^a^	NA	−12.4
Red blood cell count	F	E2	4170	3327	1.25	8560	6834	1.25	−13.0
Hematocrit	M	E4	601	247	2.43	2090	NA	NA	−11.7
Hematocrit	F	E2	5430	4178	1.30	11100	8582	1.29	−10.1
Hemoglobin	M	E4	872	227	3.84	NA	NA	NA	−8.59
Hemoglobin	F	E2	4840	3889	1.24	9930	7989	1.24	−11.3
Platelet count	M	E4	156	36.4	4.29	539	NA	NA	12.0
Red cell distribution width - SD	F	E3	8370	5756	1.45	10700	9281	1.15	11.1
**Clinical chemistry**									
Chlolesterol	M	E4	177	91.4	1.94	365	189	1.93	81.7
Chlolesterol	F	E4	640	387	1.65	1330	813	1.64	53.8
Triglycerides	M	E4	12.5	7.26	1.72	26.2	15.2	1.73	−62.5
Total protein	M	E4	1080	575	1.88	2970	1629	1.82	14.2
Glucose	M	E2	866	632	1.37	1780	1297	1.37	−35.4
Glucose	F	E2	4450	2648	1.68	9140	5440	1.68	−12.2
Alkaline phosphatase	M	E2	2300	1393	1.65	4730	2861	1.65	−15.4
Alkaline phosphatase	F	E4	308	136	2.27	701	309	2.27	−25.7
Albumin	M	E2	6750	4696	1.44	13200	9174	1.44	5.49
Potassium	F	E2	7900	5086	1.55	16200	10450	1.55	−7.07
Total Bilirubin	F	E4	19.4	3.77	5.14	48.2	8.98	5.37	−18.2
**Thyroid**									
Thyroid gland weight	M	E2	1030	720	1.43	2010	1407	1.43	41.9
Thyroid gland weight	F	E2	3010	1698	1.77	6190	3488	1.77	−17.6
Relative thyroid gland weight	M	E2	958	710	1.35	1870	1388	1.35	45.7
Relative thyroid gland weight	F	E2	3220	1759	1.83	6610	3614	1.83	−16.5
Serum free T4 (S-fT4)	M	E4	158	117	1.35	328	243	1.35	−67.2
Serum free T4 (S-fT4)	F	E2	821	683	1.20	1690	1402	1.21	−50.7
Serum free T3 (S-fT3)	M	E4	428	196	2.18	976	449	2.18	−25.0
Serum free T3 (S-fT3)	F	E2	2280	1496	1.52	4680	3072	1.52	−22.5
Serum TSH^c^	M	E4	0.29	0.04	6.50	0.58	0.09	6.50	5450
Serum TSH^c^	F	E2	206	126	1.63	402	246	1.63	1355
Large thyroid follicles (%)	F	E4	105	52.7	1.99	218	110	1.99	−70.9
**Gonadotropins**									
Serum LH	M	E2	1160	648	1.79	2390	1331	1.80	−27.8
Serum FSH	M	E2	1600	1116	1.43	3290	2292	1.44	−21.1
**Retinoids**									
Liver retinol concentration	M	E4	31.2	18.8	1.66	66.6	40.0	1.66	−44.6
Liver retinol concentration	F	E2	1000	762	1.31	2060	1565	1.32	−43.9
Liver retinyl esters concentration	M	E4	108	60.1	1.80	225	126	1.79	−63.4
Liver retinyl esters concentration	F	E2	793	670	1.18	1630	1375	1.19	−51.9
Liver all-*trans* RA concentration	M	E2	1920	1143	1.68	3750	2233	1.68	20.7
Liver 9*c*-4o-13,14-dh-RA concentration	F	E4	55.5	39.6	1.40	115	82.1	1.40	−78.2
Liver retinol amount	M	E2	1860	1111	1.67	3820	2282	1.67	−18.5
Liver retinol amount	F	E2	2840	1476	1.92	5820	3032	1.92	−18.5
Liver retinyl esters amount	M	E2	620	525	1.18	1270	1078	1.18	−45.7
Liver retinyl esters amount	F	E2	1620	1206	1.34	3300	2477	1.33	−30.1
Liver all-*trans* RA amount	M	E2	95.1	36.8	2.58	196	76	2.58	92.7
Liver 9*c*-4o-13,14-dh-RA amount	F	E4	59.6	37.4	1.59	124	77.9	1.59	−70.3
Kidney retinol concentration	M	E2	863	640	1.35	1690	1250	1.35	51.9
Kidney retinol concentration	F	E2	2060	1346	1.53	4030	2630	1.53	30.7
Kidney retinyl esters concentration	M	E4	42.2	13.6	3.10	85.2	27.5	3.10	256
Kidney all-*trans* RA concentration	F	E2	2930	2052	1.43	5720	4009	1.43	20.7
Kidney retinol amount	M	E2	897	638	1.41	1750	1244	1.41	49.0
Kidney retinol amount	F	E2	2240	1398	1.60	4370	2731	1.60	28.0
Kidney retinyl esters amount	M	E4	38.8	12.5	3.11	78.3	25.2	3.11	253
Kidney all-*trans* RA amount	F	E2	3290	2092	1.57	6430	4088	1.57	18.2
Serum retinol concentration	F	E2	2470	1521	1.62	4820	2970	1.62	25.0
**Bone densitometry**									
Cortical area of diaphysis	M	E2	5930	3506	1.69	12200	7202	1.69	−6.19
Trabecular area of metaphysis	F	E2	6230	3714	1.68	12200	7255	1.68	9.25
**Bone biomechanics**									
F yield	F	E2	2860	1647	1.74	5880	3384	1.74	−18.3
**Organ weights**									
Liver weight	M	E4	42.3	19.8	2.13	88.4	41.3	2.14	65.0
Liver weight	F	E4	512	263	1.95	1090	565	1.93	43.1
Relative liver weight	M	E4	61.0	34.8	1.75	127	72.4	1.75	66.0
Relative liver weight	F	E4	552	359	1.54	1170	766	1.53	44.8
Relative thymus weight	M	E2	2230	1270	1.76	4350	2480	1.75	17.6
Ovaries weight	F	E4	201	31.7	6.34	510	79.6	6.41	17.0
Relative ovaries weight	F	E5	661	67.5	9.79	826	164	5.04	16.0
**DNA damage markers**						CES 100%			
Liver p53	F	E4	65.2	30.0	2.18	2240	1053	2.13	141
Liver p53 Ser15	F	E5	361	124	2.91	438	411	1.07	181
Liver pChk2 Thr68	F	E5	337	98.2	3.43	382	165	2.31	185
Liver enzymes^d^									
EROD activity	M	E4	1.41	0.850	1.66	2.82	1.71	1.65	
EROD activity	F	E5	33.6	15.8	2.13	57.0	29.6	1.93	
PROD activity	M	E5	3.87	2.74	1.41	5.49	4.04	1.36	
PROD activity	F	E5	26.4	13.5	1.96	36.5	20.2	1.81	
CYP2B1 mRNA	M	E5	1.52	0.584	2.60	2.15	0.895	2.40	
CYP2B1 mRNA	F	E5	12.4	5.74	2.16	15.5	7.76	2.00	
CYP3A1 mRNA	M	E4	5.88	3.63	1.62	11.8	7.26	1.63	
CYP3A1 mRNA	F	E4	6.75	3.19	2.12	13.5	6.39	2.11	
UGT1A1 mRNA	M	E2	175	134	1.30	342	262	1.31	
UGT1A1 mRNA	F	E2	423	305	1.30	826	596	1.39	
UGT1A6 mRNA	M	E4	15.6	8.99	1.74	31.3	18.0	1.74	
UGT1A6 mRNA	F	E4	73.8	37.8	1.95	149	76.42	1.95	

a Not available.

b Calculated as the percent difference between controls and high dose according to the fitted model.

c Half min value added to zeros.

d Enzyme induction data from Roos *et al.*, 2011.

### In life observations

There was no mortality. Body weight development was retarded at 1700 mg/kg bw and slightly also at 1000 mg/kg bw in both genders during the loading dosing, but recovered completely by the end of the study (Fig. 1). Due to unexpected decrease in body weight at the highest dose the third loading dose was replaced with corn oil vehicle. The top dose (1700 mg/kg bw) animals showed slightly and transiently reduced activity during loading dosing. Feed consumption was temporarily reduced in males maximally by about 25% at the two highest doses and in females maximally by about 20% at the three highest doses during loading dosing, but recovered thereafter. Water consumption was unaffected (data not shown).

### Adipose tissue PCB 180 concentrations

**Figure 2:**
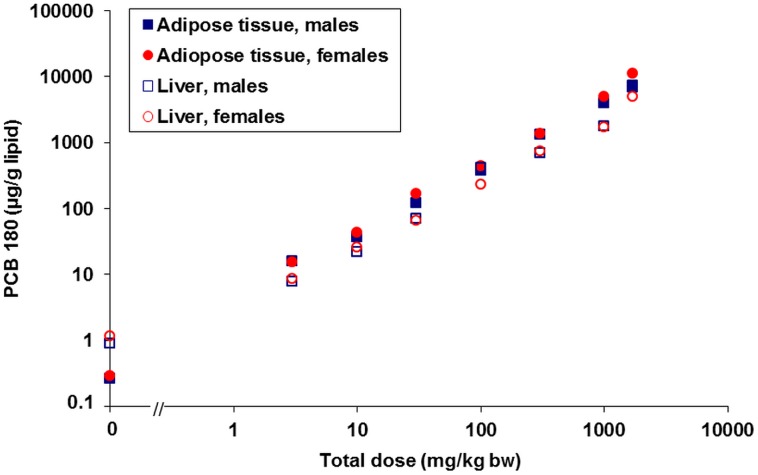
Lipid based adipose tissue and liver PCB 180 concentrations at the end of the study. Pooled samples of treatment groups (5 individuals per pool), log-log scale. Liver data from Roos *et al.*, 2011 [17]. PCB 180 tissue concentrations reflected accurately the administered doses.

Background adipose tissue PCB 180 concentrations in control rats at the end of the study were within the range of human background levels, indicating lack of contamination in the animal room. PCB 180 concentrations reflected accurately the administered doses (Fig. 2, Table S1). Females had higher adipose tissue concentrations than males, especially at the two highest dose levels, but liver concentrations were more similar. For comparison, liver concentration data [17] are also shown. Overall, the lipid based adipose tissue concentrations were slightly (up to two times) higher than liver concentrations, but on dry weight basis the difference was an order of magnitude or even more.

### Open field test for spontaneous locomotor activity

**Figure 3:**
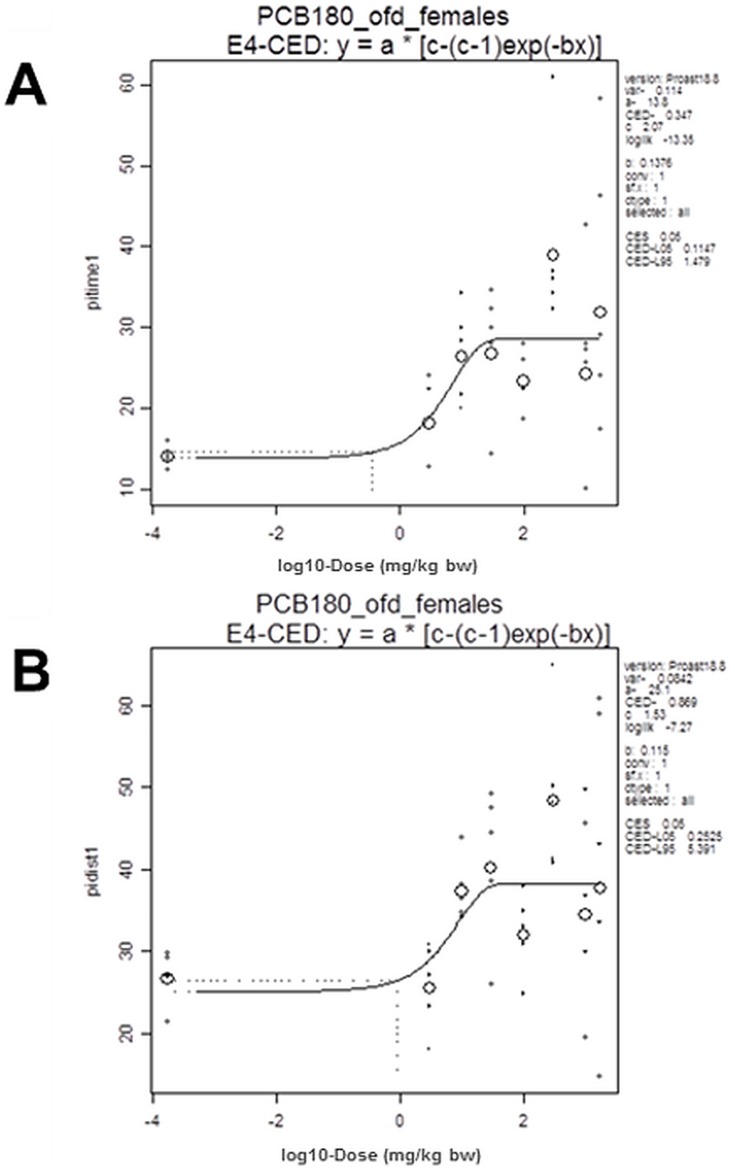
BMD analysis of percentage of time (A) and distance moved (B) in the inner zone of open field. Time and distance in the inner zone were dose-dependently increased in females on study day 24 (1^st^ test day). Small symbols indicate individual samples, large circles the group mean; the vertical dotted line indicates the dose (CED) with 5% increase (CES -0.05) compared to background (a parameter).

Distribution of activity between the inner and outer zones of the open field was significantly affected by PCB 180 only in females (p<0.05). On study day 24 (1^st^ test day), there were dose-related increases in percentage of time and distance moved in the inner zone (CED 0.35 mg/kg bw, 1.55 µg/g lipid and CED 0.87 mg/kg bw, 4.12 µg/g lipid, respectively, Tables 2 and 3, Fig. 3) and, conversely, decreases in percentage of time and distance in the outer zone of the open field. These differences ameliorated across the five days of testing, as demonstrated by significant interactions between exposure and test days for both measures (percentage of time in inner zone - p<0.05; percentage of distance moved in inner zone - p<0.01), indicating differences in habituation between groups. As a consequence, dose-response relations were no longer significant on day 28. To quantify effects on habituation, the mean of time in inner zone across test days 2–5 was divided by time in inner zone on day 1. This revealed a dose-dependent decrease (CED 184 mg/kg bw, 1130 µg/g lipid). A similar calculation for habituation of the percentage of distance moved resulted in dose-related decreases with a CED 216 mg/kg bw (1340 µg/g lipid). Trend analysis of total distance moved (sum of activity in both zones) revealed a quadratic relation to dose across all test days (p<0.05) and on each of the days 2–5 (p<0.05), with elevated activity values at intermediate dose levels compared to controls and the top dose group. There were no clear-cut differences in total distance moved between test days, irrespective of exposure. Thus, total activity did not habituate across the days of measurement. No significant dose-response relations were found in exposed males.

### Hematology

**Figure 4:**
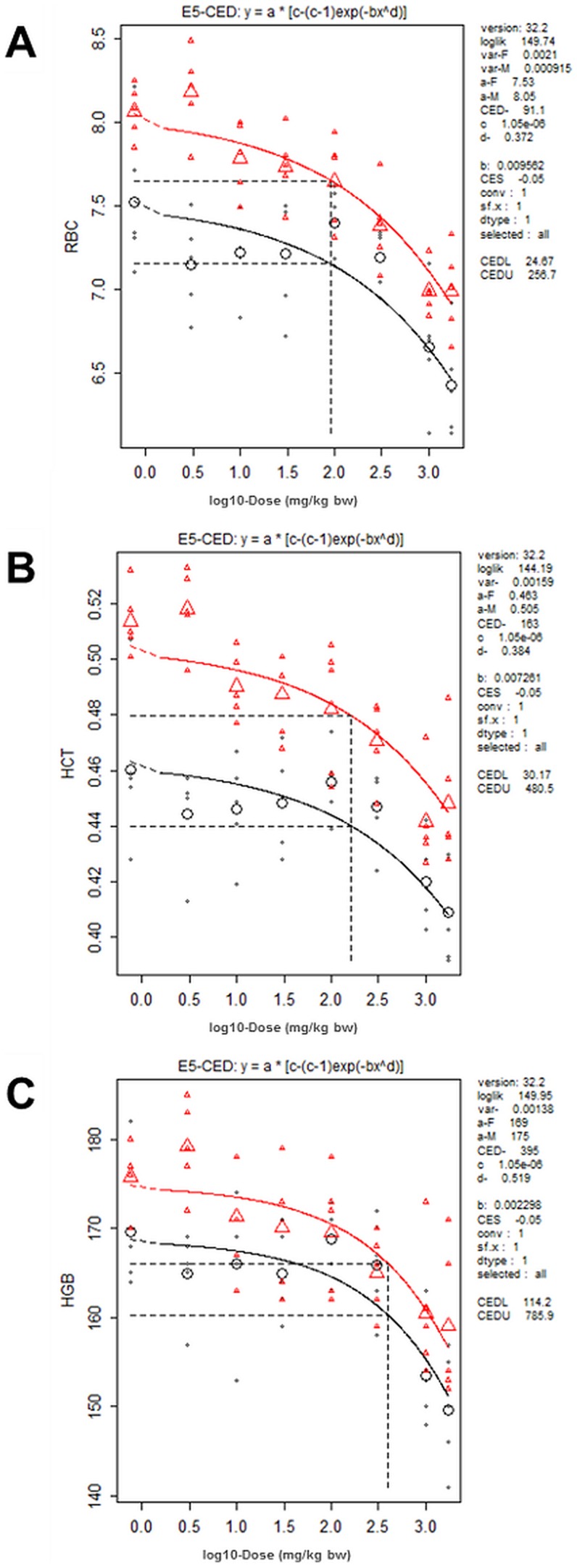
BMD analysis of red cell count (A), hematocrit (B) and hemoglobin (C) in males (triangles) and females (circles). These parameters were dose-dependently decreased in both genders. Small symbols indicate individual samples, large symbols the group mean; the vertical dotted line indicates the dose (CED) with 5% decrease (CES -0.05) compared to background (a parameter). (Optimal models used for CED calculations as shown in Table 2 were determined separately for females and males and are not necessarily the same shown here).

Results of hematological analyses are shown in Table S2. The characteristic feature was significant and dose-related decreases in the amount of red blood cells (RBC, HCT) and blood HB in both genders. Males were more sensitive and the lowest CED was for RBC, 138 mg/kg bw (598 µg/g lipid) for males and 638 mg/kg bw (4170 µg/g lipid) for females (Fig. 4, Tables 2 and 3). MCV was not affected in males, but showed an increasing trend in females. MCH and MCHC were not affected in females, but an increasing trend was observed in males. As these trends were within normal range of variation the observed effect can be regarded as normocytic and normochromic anemia, and only red blood cells were affected.

### Clinical chemistry

Results of clinical chemistry analyses are shown in Table S3. Serum cholesterol levels were significantly increased both in males (CED 38.3 mg/kg bw, 177 µg/g lipid) and in females (CED 192 mg/kg bw, 640 µg/g lipid) (Tables 2 and 3). In males, serum triglyceride levels were significantly decreased (CED 3.31 mg/kg bw, 12.5 µg/g lipid) and total protein levels increased at ≥1000 mg/kg bw (CED 255 mg/kg bw, 1080 µg/g lipid). In females, serum ALP activity was slightly, but significantly decreased at the two highest dose levels (CED 67.6 mg/kg bw, 308 µg/g lipid) [17].

### Thyroid hormones

**Figure 5:**
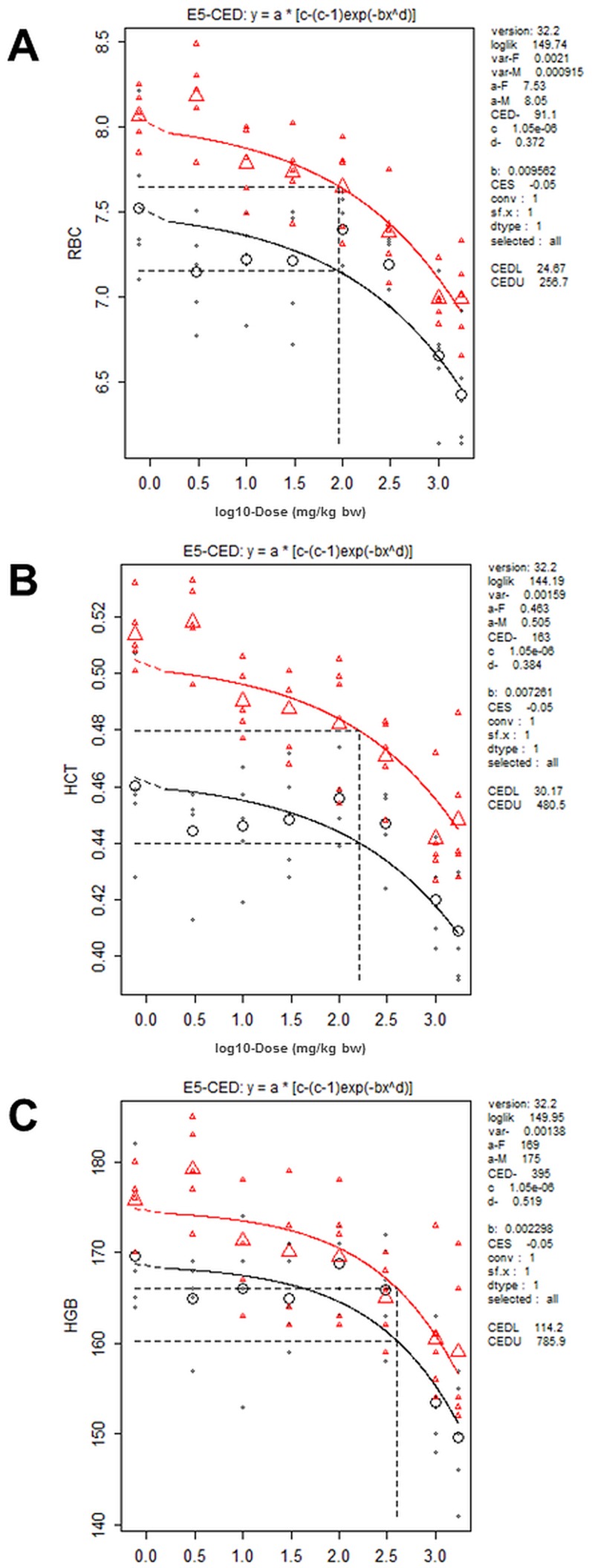
BMD analysis of serum free T4 (A), serum free T3 (B) and serum TSH (C) in males (triangles) and females (circles). T4 and T3 were dose-dependently decreased in both genders, and TSH increased in males only. Small symbols indicate individual samples, large symbols the group mean; the vertical dotted line indicates the dose (CED) with 5% decrease or increase (CES -0.05) compared to background (a parameter). (Optimal models used for CED calculations as shown in Table 2 were determined separately for females and males and are not necessarily the same shown here).

Serum levels of free T4 and free T3 were dose-dependently decreased in both genders males being more sensitive. CED values for T4 were 38.1 mg/kg bw (158 µg/g lipid) and 128 mg/kg bw (821 µg/g lipid) in males and females, respectively, and CED for T3 290 mg/kg bw (428 µg/g lipid) and 354 mg/kg bw (2280 µg/g lipid), in males and females, respectively (Fig. 5, Tables 2, 3 and S4). The decrease in free T3 was modest, and reached statistical significance only in males at 1000 mg/kg bw. Serum TSH levels showed an increasing trend in males (CED 0.07 mg/kg bw, 0.29 µg/g lipid), but due to high within group variability the differences between controls and treated groups did not reach statistical significance.

All four potential mono-hydroxyl metabolites of PCB 180 were able to displace T4 from TTR. Average IC_50_ values (n = 2) ranged from 13.0 to 19.6 nM, with 4′-OH-PCB 172 being the most potent competitor. Compared to the natural ligand T4, the tested OH-PCBs had relative potencies 3.1 to 4.6 times higher for TTR binding. Dose-response curves and IC50 and relative potency values of the four mono-hydroxyl PCB metabolites are given in Table S13.

### Steroids and gonadotropins

In females, serum estradiol levels showed only some non-significant decreases at the 3 highest dose groups, but no clear changes were seen in serum progesterone or LH levels (Table S5). In males, serum testosterone levels were non-significantly decreased at the highest dose only. A significant decreasing trend was observed in serum LH and FSH levels, the latter being significantly below controls at 1700 mg/kg bw (CED 266 and 358 mg/kg bw [648 and 1116 µg/g lipid] for LH and FSH, respectively).

### Retinoids

Tissue retinoid concentrations are shown in Table S6, and liver and kidney retinoid amounts in Table S7. Liver retinol concentrations were dose dependently decreased in both males and females with CED of 32 and 156 mg/kg bw (31 and 1000 µg/g lipid), and max. decreases of 47 and 43%, respectively (Tables 2, 3 and S6). Liver retinyl ester concentrations were also dose-dependently decreased in both males and females with CED of 22 and 122 mg/kg bw (108 and 793 µg/g lipid) and max. decreases of 63 and 52%, respectively. Dose-dependently increased liver concentrations of all-*trans* RA were only seen in males (CED 450 mg/kg bw, 1920 µg/g lipid, max. increase 20%), while liver concentrations of 9*c*-4o-13,14-dh-RA were reduced only in females (CED 12 mg/kg bw, 56 µg/g lipid, max. decrease 78%). Corresponding results were obtained for the total retinoid contents of liver and kidneys (Table S7). Concentrations of 9-*cis* RA and 13-*cis* RA were below the detection limit in all liver samples.

Kidney retinol concentrations were dose-dependently increased in both males and females (CED 200 and 335 mg/kg bw [863 and 2060 µg/g lipid], max. increases 52 and 28%, respectively (Tables 2, 3 and S6). Kidney retinyl ester concentrations were dose-dependently increased only in males (CED 9.9 mg/kg bw, 42 µg/g lipid, max. increase of 254%), while kidney all-*trans* RA concentrations were dose-dependently increased only in females (CED 457 mg/kg bw, 2930 µg/g lipid, max. response 20%). Corresponding results were obtained for the total retinoid contents of the kidneys (Table S7). Concentrations of 9-*cis*-RA, 13-*cis*-RA and 13-*cis*-4o-RA were below the detection limit in all kidney samples.

Serum retinol concentrations were dose-dependently increased in females only (CED 400 mg/kg bw, 2470 µg/g lipid, max. increase 23%). Serum retinyl ester concentrations were not affected (Tables 2, 3 and S6). Concentrations of 9-*cis* RA, 13-*cis* RA and 13c-4o-RA were below the detection limit in all serum samples.

### p53 and DNA damage markers

**Figure 6:**
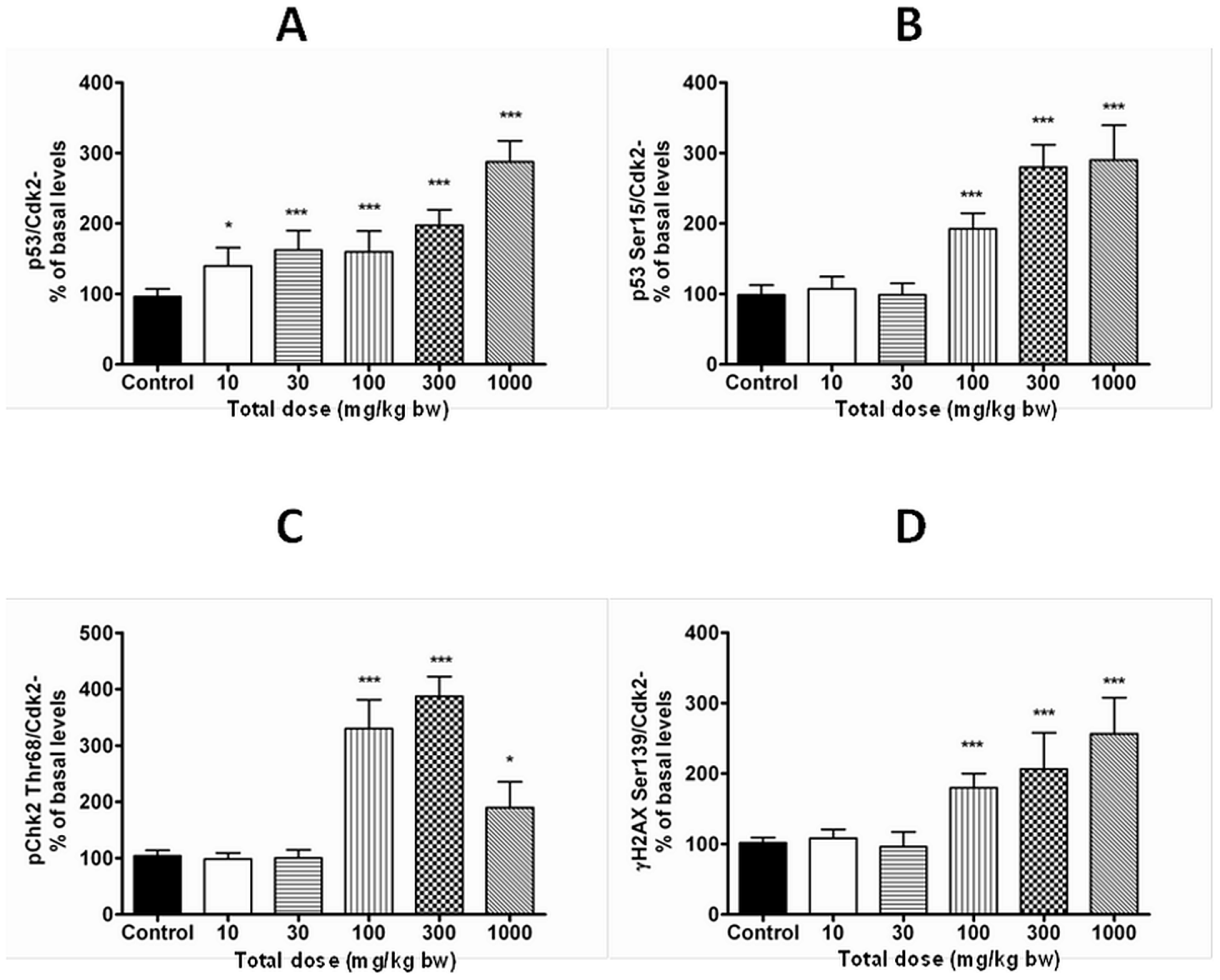
Densitometric analysis of Western blots for total p53 (A), p53 Ser15 (B), pChk2 Thr68 (C) and γH2AX Ser139 (D) protein in livers of females. Tumor suppressor protein p53 and the DNA damage signaling markers were dose-dependently increased only in females. Each column represents mean ± SD (n = 5) as percent of control after adjustment to the loading control (Cdk2). Data was obtained from at least three independent analyses.

Expression of the tumor suppressor protein p53 was dose-dependently increased in livers of female rats (CED 472 mg/kg bw, 2240 µg/g lipid) (Fig. 6, Tables 2 and 3), but no changes were observed in expression of p53 regulating pMdm2 Ser166 or markers of activated protein kinase B/extracellular-regulated kinases (Akt/Erk) signaling Akt Ser473and Erk Tyr204 (data not shown). The ability of PCB 180 to activate DNA-damage signaling was studied by analyzing the expression of p53 Ser15, γH2AX Ser319 and pChk2 Thr68. These markers were dose-dependently increased at 100 mg/kg bw and above with CED (100%) of 101 mg/kg bw (438 µg/g lipid) for p53 Ser15, and 81.1 mg/kg bw (382 µg/g lipid) for pChk2 Thr68 (Fig. 6, Tables 2 and 3; CED for γH2AX Ser319 could not be determined). None of these markers were changed in livers of males.

### Organ weights

Absolute organ weights are shown in Table S8. Liver weights were dose-dependently increased in both genders, more in males than in females, with CED values of 11.6 and 225 mg/kg bw (42.3 and 512 µg/g lipid) and max. increases of 66 and 45%, respectively (Tables 2 and 3) [17]. Thyroid weights were dose-dependently increased in males (CED 239 mg/kg bw [1030 µg/g lipid], max. increase 17%), but decreased in females (CED 443 mg/kg bw [3010 µg/g lipid], max. decrease 18%). Ovary weights were increased with CED of 46.8 mg/kg bw [201 µg/g lipid] and max. increase of 17%. It is important to note that thymus weight, the sensitive indicator for exposure to dioxin-like compounds, was not decreased.

### Histopathology

In the liver, a dose-related increase in incidence and severity of centrilobular hypertrophy was observed in males and females with CED of 14.8 and 205 mg/kg bw, respectively, for mild hypertrophy [17]. Males were more sensitive both in terms of CED and severity.

**Figure 7:**
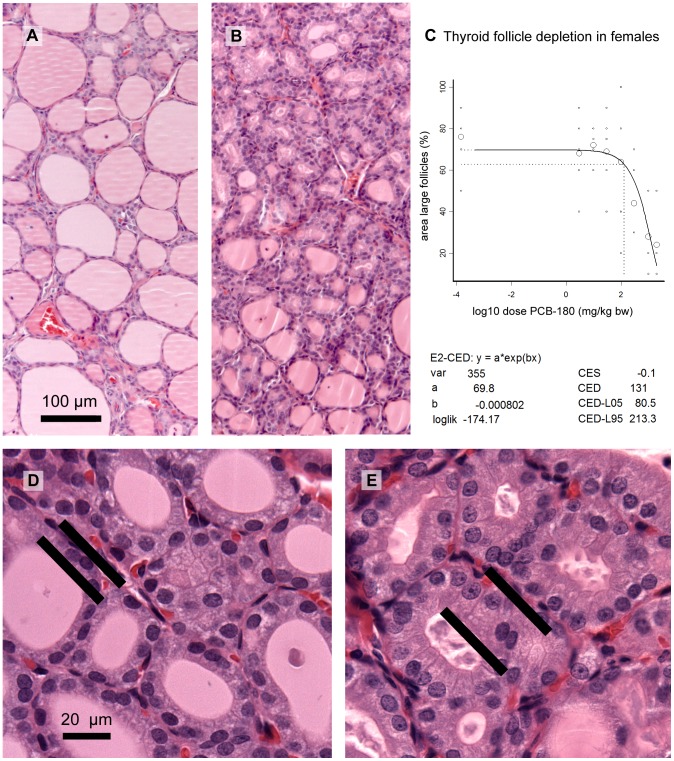
Microphotographs of the thyroid gland of female rats and BMD analysis of follicle depletion. Decrease of follicle contents is illustrated by comparing samples from controls (A) and 300 mg/kg bw (B). BMD analysis of follicle depletion (measured as the estimated area with large follicles on representative sections at low power magnification) indicated that this effect was dose-dependent (C). Small circles indicate individual samples, large circles group means; the vertical dotted line indicates the dose (CED) with 5% decrease (CES -0.05) compared to background (a parameter). The thyroid glands also showed follicle epithelium hypertrophy as illustrated by comparing 10 (D) and 300 mg/kg bw (E). Epithelium cell height is indicated by bars at basal and apical cell borders.

In the thyroid gland, there was a dose-related decrease of the area of large follicles in females (Fig. 7; CED 131 mg/kg bw, 105 µg/g lipid), indicating depletion of follicle contents. In control males the proportion of large follicles was much lower than in control females (comparable with high dose females), and the area of large follicles was not affected by the treatment. At high magnification, a dose-dependently increasing hypertrophy of follicle epithelial cells was observed in females (Fig. 7). Males had a higher basal score for hypertrophy, and no significant treatment-related increase.

**Figure 8:**
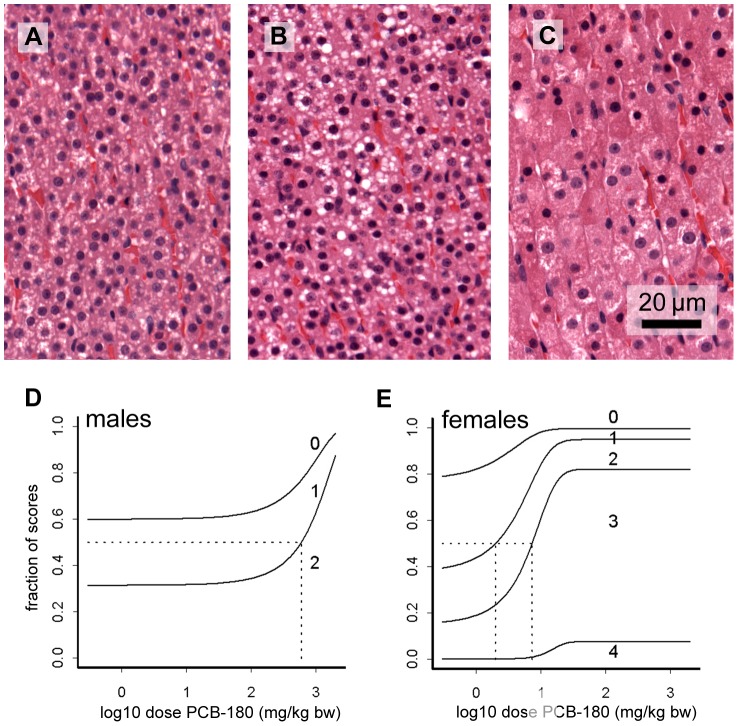
Microphotographs of the adrenal cortex and BMD analysis of *zona fasciculata* activation. Two stages of activation of *zona fasciculata* are shown in comparison with no activation: A, no activation (stage 0, 0 mg/kg bw); B, moderate activation with vacuolization and slight hypertrophy (stage 2, 10 mg/kg bw); C, strong activation with severe hypertrophy (stage 4; 300 mg/kg bw). This staging system was used for semi-quantitative assessment of the population, as shown in the BMD graphs for males (D) and females (E). In these graphs, the lines separate the fractions of the population with the indicated stages of activation, e.g. in control males, similar fractions are at stage 0, 1 and 2, whereas at the highest dose, most samples are at stage 2. Vertical dotted lines show the dose at which the average animal (horizontal dotted line) progresses to a further stage of activation.

In the adrenal cortex, cells of *zona fasciculata* showed activation as indicated by hypertrophy and vacuolization (Fig. 8). Semi-quantitative staging of hypertrophy revealed dose-dependent responses. Females were more sensitive showing progression of hypertrophy to further stages compared to males, and they also had a lower CED (2.0 mg/kg bw) than males (594 mg/kg bw). Similar to the *zona fasciculata*, there was hypertrophy and vacuolization in cells of the z*ona reticularis* with a significant dose response in females. The inner zones of the cortex occasionally also showed hyperemia with a significant dose response in females but not in males. The CEDL for this effect in females was 2526 mg/kg bw.

**Figure 9:**
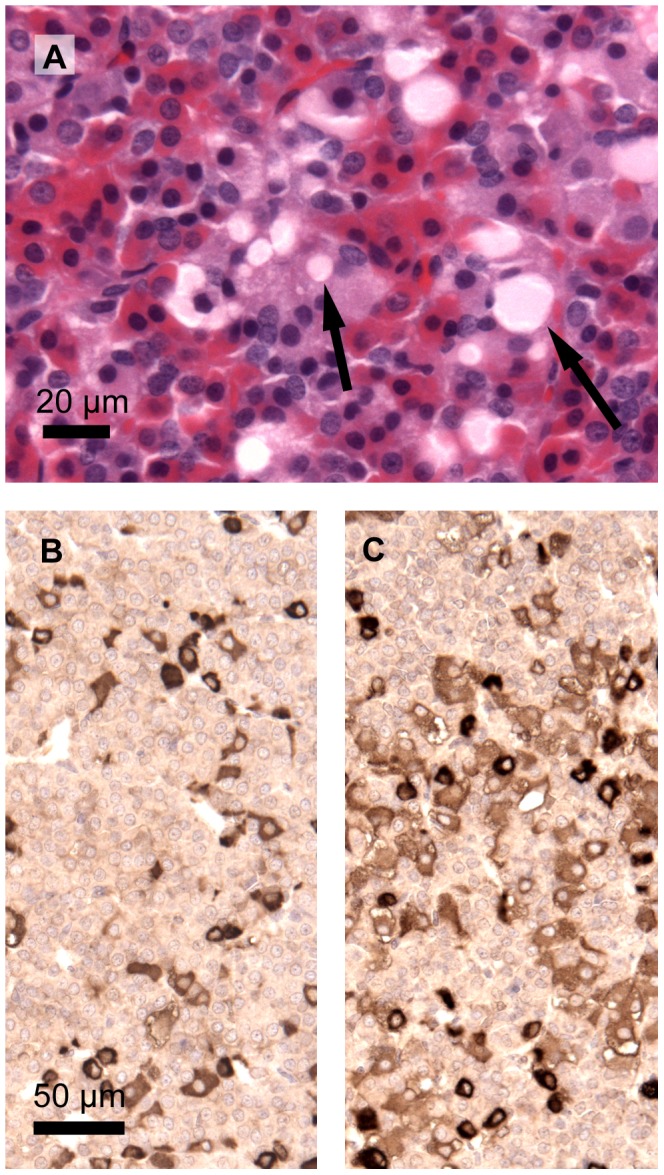
Microphotograph of frontal lobe of the pituitary of males. A male at the top dose male (A; 1700 mg/kg bw) showing vacuoles (left arrow) or extracellular deposits (right arrow). The contents of these vacuoles/deposits are not unambiguously identifiable with immunostaining for TSH or ACTH. Immunostaining of the frontal lobe of the pituitary, however, shows more TSH positive cells at the top dose (C, 1700 mg/kg bw) compared to controls (B).

In the pituitary, there were vacuoles or extracellular deposits in the frontal lobe of the top dose animals in 4/5 males and 2/5 females (Fig. 9). To assess whether these cysts resulted from hyperproduction and/or -secretion of pituitary hormones, immunohistochemical detection of TSH and ACTH was performed. Only occasionally slight staining for TSH in some of these vacuoles/deposits, and a few moderately staining vacuoles/deposits with ACTH in two male samples were observed. In males, the density of TSH positive cells appeared to be higher in top dose samples compared to controls (Fig. 9).

No exposure related overt effects were observed in the pancreas, including Langerhans islets, the testis, prostate, epididymis, seminal vesicle, thymus, spleen, ovary, mammary gland and kidney. Morphometric analysis of thymus of control and top dose males confirmed the lack of an effect by histological reading (cortex/medulla ratio was 3.5±1.3 in controls and 3.0±0.7 in top dose samples).

### Sperm analyses

Cauda epididymal sperm density analyzed for control and high dosage (1700 mg/kg bw) males were not affected by the treatment (Table S9).

### Bone geometry, densitometry and biomechanics

None of the examined bone morphological or densitometry parameters were dose dependently altered according to one-way ANOVA (Table S10). However, using the benchmark dose approach a dose dependent decrease of the cortical area of tibial diaphysis was observed in males with a CED of 1380 mg/kg bw (5930 µg/g lipid) and max. decrease of 6.5% (Tables 2 and 3). In females a dose dependent increase of the trabecular area of metaphysis was observed with a CED of 1040 mg/kg bw (6230 µg/g lipid) and max. increase of 8.2% (Tables 2 and 3).

Biomechanical testing with three-point bending test of tibial shaft revealed decreased yield force in females (CED 476 mg/kg bw, 2860 µg/g lipid, max. decrease 18%; Tables 2, 3 and S10). This indicates that less force was needed to reach plastic deformation where cracking is initiated and bone starts to break.

### Brain amino acid analyses

Amino acid concentration in cerebrum did not show significant differences between controls and PCB 180 exposed groups (Table S11). However, glutathione concentration showed a significant decreasing trend (p = 0.037) in males with a max. decrease of 18% at 1000 mg/kg. The likely explanation for decreased glutathione levels is PCB 180 -induced oxidative stress [42].

### Brain dopamine and nicotinic receptor analyses

No significant differences between controls and the 1000 mg/kg bw dosage group were found in the specific [^3^H]SCH23390 binding to the D1/D5 dopamine receptors in cerebrum (Table S12). Similarly, comparison of the specific binding of [^3^H]epibatidin to the high or low affinity sites on the nicotinic receptor subunit α4/β2 showed no significant difference.

## Discussion

The present study is the first report on comprehensive toxicological profile of the major indicator PCB 180. Use of ultrapure test compound made it possible to examine the effects of PCB 180 without contribution of DL impurities. The dose-levels were selected to cover the whole spectrum of biological effects from subtle induction of xenobiotic metabolism to clear toxicity. Because of the loading dose/maintenance dose protocol the adipose tissue PCB 180 concentrations at the end of the study represent the kinetic steady state and make it possible to relate the observed effects to the internal dose. Furthermore, by using internal dose at steady state it is possible to directly compare and extrapolate tissue levels associated with observed effects to corresponding human/wildlife situations.

PCB 180 showed a phenobarbital type of induction of xenobiotic metabolism consistent with induction of CYP2B1 and UGTs 1A1 and 1A6, likely due to activation of the constitutive active (androstane) receptor (CAR) [17]. Recently, pronounced CAR activation along with minor effects on the pregnane-X-receptor (PXR) were reported in rat hepatocytes treated with various NDL-PCBs, including PCB 180 [43]. Lack of the typical AHR dependent responses on hepatic CYP1A1 induction [17], thymus weight and histology, as well as body weight development confirm that PCB 180 lacks several of the specific effects required for assignment of a toxic equivalency factor (TEF) for DL compounds according to the WHO [44].

### PCB tissue concentrations

The lipid based adipose tissue PCB 180 concentrations in the exposed animals ranged from 15.5 µg/g lipid at a total dose of 3 mg/kg bw up to 11 300 µg/g lipid at 1700 mg/kg bw (Fig. 2, Table S1). For comparison, in the WHO mother's milk survey carried out in 2001–2002 the range of PCB 180 concentrations was 0.006–0.337 µg/g lipid (median 0.046 µg/g lipid) [1], [2]. The same figures for the sum of PCBs were 0.045–1.37 µg/g lipid (median 0.272 µg/g lipid). In Baltic fishermen the range of PCB 180 concentrations was 0.19–1.2 µg/g lipid (median 0.460 µg/g lipid) and that for the sum of PCBs 0.950–8.700 µg/g lipid (median 2.70 µg/g lipid) [45]. Thus, the lowest dose level of this study resulted in 13-fold higher PCB 180 adipose tissue concentration than the maximum value in the Baltic fisherman cohort, but if the comparison is made to the maximum concentration of the sum of PCBs, the difference is only 1.8-fold. Comparison with the median values of the WHO mother's milk and the Baltic fisherman cohorts reveals 337- and 34-fold difference for PCB 180 and 57- and 5.7–fold difference for the sum of PCBs. Thus, the lipid based tissue concentrations of the rats were clearly above the general population levels, however close to the levels in highly exposed human populations.

### In-life observations and behavioral effects

Daily loading dosing at the two highest dose levels resulted in transiently reduced feed consumption and activity, and retarded body weight development that subsided on transition to the weekly maintenance dose schedule. In previous studies with NDL-PCBs 128 and 153 [7], [8], [46] no effects on body weight or feed intake were reported, most likely because the daily doses were much lower. In the present study the daily dose during loading dosing was 288 mg/kg bw, whereas for example in the study of Chu et al. [6] the estimated daily dose of PCB 153 (given in diet) was only 4.13 mg/kg bw. The observed transient alterations are clearly different from the more permanent wasting syndrome induced by DL compounds [47], and considering the long elimination half-life of PCB 180 [14], [15] the likely explanation is a local effect on the GI tract.

Altered locomotor activity of female rats was observed in the open field conducted during study days 24–28. This effect was mainly expressed on distribution of activity, namely, increases in distance moved and time in the inner zone. The changes were present only on the first day of testing, showing quick habituation thereafter which resulted in a similar distribution of activity in all groups by the end of the testing period. On the other hand, total activity in both zones was slightly elevated only in intermediate dose groups compared to controls and the top dose group, an effect which did not habituate. Altogether, these findings suggest an effect on emotional responses to an unfamiliar environment in exposed females, together with impaired behavioral inhibition. In contrast to behavioral alterations, amino acid concentrations or receptor binding at dopamine D1/D5 receptors and nicotinic receptors were not affected in whole cerebrum. Spontaneous locomotor activity is a highly integrative behavior, which as such may be affected by different chemicals and via different mechanisms. Altered locomotor activity is among the most frequently reported behavioral effects after exposure to single PCB congeners and mixtures in different species (reviewed in [48]). Frequently, sensitivity differences have been reported between genders. Also, impaired response inhibition has been observed in PCB exposed male and female Long Evans rats [49], male monkeys (*Macaca fascicularis*) [50] and human children [51]. A similar effect as detected here for PCB180 has been described previously for NDL-PCB 47 and DL-PCB 77 after *in utero* and lactational exposure in rats [52] and after developmental exposure to Aroclor 1254 in female mice [53]. Furthermore, increased locomotion was detected after subacute exposure to Aroclor 1254 in mice, together with elevated dopamine concentration in the striatum and loss of dopaminergic neurons in the midbrain [54]. The failure to find an effect on dopamine receptors in our study does not exclude possible PCB 180-induced changes in neurotransmitter levels. Further experiments should include analyses of dopamine concentrations and binding related to the D2 receptor family in a region-specific approach. The dopaminergic system appears to be critically involved in the etiology of attention deficit hyperactivity disorder (ADHD) [55], [56] and PCB exposure was shown to affect behavioral domains that are altered in children suffering from ADHD [57], [58]. The present study showed that the alteration of activity is in fact the most sensitive effect of PCB 180 (CED 0.35 mg/kg bw, 1.55 µg/g lipid) observed after exposure of young adult female rats. The same batch of ultrapure PCB 180 was shown to induce increased consumption of sweetened solution in female offspring [59] and impaired learning in both genders of rat offspring [60] after *in utero*/lactational exposure, and to alter response rate on an operant conditioning task [61] after neonatal exposure.

### Hematology and clinical chemistry

Dose-dependently and significantly decreased number of red blood cells (decreased RBC and HCT) was observed at the three highest dose-levels of PCB 180 in males and two highest dose-levels in females. This change was associated with decreased blood HB concentration, although with slightly higher CED. Due to characteristics of normochromic and normocytic anemia the likely reasons are decreased erythropoiesis and increased hemolysis. Because serum bilirubin levels were not increased, hemolysis is not likely to play a significant role. Similar decreases in number of red blood cells and blood HB have been reported in previous 13-week studies with Sprague-Dawley rats after relatively high doses of mono-ortho PCB 105 [62], DL-PCB 126 [63] and PCDDs [64], [65].

Serum cholesterol levels were dose-dependently and significantly increased in both genders. This is also in accordance with earlier findings after treatment with mono-ortho PCB 105 [62] and DL-PCB 126 [63], and therefore increased serum cholesterol seems to be a common effect of DL- and NDL-PCBs. Males were more sensitive than females, and serum triglyceride levels were decreased only in males. Overall, changes in hematological and clinical chemistry parameters take place at high exposure levels; among these changes CED for decreased serum triglycerides in males is exceptionally low (CED 3.31 mg/kg bw, 12.5 µg/g lipid).

### Thyroid hormones and thyroid gland

PCB 180 exposure was accompanied by a whole variety of effects in the thyroid system ranging from decreased levels of circulating thyroid hormones to altered thyroid gland weight and histology, as well as increased hepatic expression (mRNA and protein) and activity of UDP-glucuronosyl transferases (UGTs), the enzymes responsible for elimination of thyroid hormones [17]. The observed effects are characteristic for DL- and NDL-PCBs as well as for PCDD/Fs [66], [67]. The best-known thyroid effect of these compounds is increased elimination of thyroid hormones subsequent to induction of UGTs. Different nuclear receptors activate the UGT isoforms responsible for thyroid hormone glucuronidation (UGT1A1 and UGT1A6), and they can be induced by DL compounds via AHR and by NDL-PCBs via CAR and PXR[17], [66], [43]. Comparison of the CED values for thyroid endpoints (Tables 2 and 3) indicates that the induction of UGTs takes place at lower exposure levels than the decrease in circulating thyroid hormones, and therefore UGT induction is a possible cause for the observed hypothyroidism. In addition, similar sensitivity difference between genders is observed both in UGT induction and decreased T4 levels, males being more sensitive (see below).

Another potentially significant mechanism of PCB-induced hypothyroidism is the displacement of thyroid hormones from their transport proteins in blood [68]. This may result in increased availability of thyroid hormones to conjugation reactions leading to enhanced elimination. Reduced transport to target cells is another possible consequence. The main transport protein in rodents is TTR. TTR plays also a significant role in human fetal brain development, because it is responsible for transport of thyroid hormones via placental and blood-brain barriers [69]. Studies for T4 displacement from TTR indicated either only a very weak potency (relative T4 potency <0.0038) [9] or modest potency (relative T4 potency 0.07) [70] for PCB 180. However, the TTR-binding potency of the PCB 180 mono-hydroxyl metabolites was 3.1–4.6 times higher than that of T4 (Table S13). These relative potency factors correspond very well with factors of 3–10 reported for other hydroxyl metabolites of PCBs in several studies [29], [71], [72]. Because TTR plays a significant role in transport of thyroid hormones through the placental and blood-brain barriers, high affinity of hydroxyl-PCBs to TTR and thyroid hormone displacement potentially results in an efficient transport and accumulation of hydroxyl-PCBs into the fetal compartment and brain at the cost of thyroid hormones [68], [73], [74]. Chemical analysis revealed dose-dependently increasing concentrations of 3′-OH-PCB 180 in livers of the rats of the present study (Al-Anati *et al.*, in preparation). At least two of the four potential hydroxyl metabolites have been found in human tissue, i.e. 3′-OH-PCB 180 in adipose tissue [75] and 4′-OH-PCB 172 in blood serum [76], [77], although the latter could also be a metabolite of PCB 170 rather than PCB 180. Based on these results, it is therefore likely that T4 displacement from TTR contributes to enhanced elimination of thyroid hormones as well as to decreased thyroid hormone levels and increased levels of PCB 180 hydroxyl metabolites in the brain.

Histopathology showed that control females have much higher proportion of large thyroid follicles than control males and that treatment with PCB 180 results in dose-dependent depletion of follicle contents only in females. This is in accordance with the following observations in males only: (1) higher basal TSH levels and dose-dependently increased circulating TSH (Table S4), (2) higher basal score for hypertrophy of the thyroid follicle epithelium, (3) increased density of immunohistochemically detected TSH positive cells in the frontal lobe of pituitary, and (4) more sensitive hepatic induction of UGT1A1 and UGT1A6 by PCB 180 [17]. These findings suggest that for compensation of decreased thyroid hormone levels, males depend on *de novo* synthesis, whereas females can use their thyroglobulin storage.

The consequences of hypothyroidism depend on the stage of development. In adult animals the signs are rather non-specific and include altered regulation of normal physiological functions and reduced rate of metabolism [78], [66]. In adult rodents low circulating thyroid hormone levels result in TSH driven overstimulation of the thyroid gland that may eventually lead to formation of thyroid tumors. In the 2-year NTP study with PCB 153 decreased serum thyroid hormone concentrations and thyroid follicular cell hypertrophy were reported, but no thyroid tumors [8]. Because of lower sensitivity of human hypothalamus-pituitary-thyroid axis this mode-of-action is not considered relevant for humans. The most adverse and permanent consequences of hypothyroidism both in animals and humans have been reported after developmental disruption of thyroid function. Developing nervous systems is particularly sensitive and even transient decrease of thyroid hormone levels may result in adverse outcome. This mode-of-action is likely to be relevant in humans [66]. In this study the observed behavioral alterations after adult exposure to PCB 180 had clearly lower CED values than the thyroid effects suggesting that these phenomena may not be interrelated.

With regard to the thyroid effects of NDL-PCBs it is important to note that in the environment they always exist together with DL compounds, and that these groups of chemicals induce UGTs via different nuclear receptor pathways (see above). Thus, there is potential for synergistic (greater-than-additive) interaction [79]. Using a mixture of 2 PCDDs, 4 PCDFs, and 12 PCBs (of which 5 NDL-PCBs) Crofton *et al.* [79] demonstrated a dose-dependent synergistic effect for T4 hypothyroidism in rats. Therefore, the potential interaction with DL compounds emphasizes the significance of thyroid hormone disruption as an endpoint of toxicity of NDL-PCBs, although based on CED values thyroid effects are not highly sensitive.

### Retinoids

Exposure to PCB 180 resulted in dose-dependently decreased hepatic retinol and retinyl palmitate levels, as well as increased renal retinol levels in both genders. In contrast, hepatic levels of all-*trans*-RA and 9c-4o-13,14-dh-RA, renal levels of retinyl palmitate and all-*trans* RA, as well as serum retinol levels were differently affected in male and female rats. As vitamin A is mainly stored in the liver in the form of retinyl palmitate, the reduction of both hepatic retinyl palmitate and retinol levels suggests increased mobilization of retinoids into the active all-*trans* RA, i.e. retinyl palmitate is hydrolyzed to retinol, which is further oxidized by alcohol and aldehyde dehydrogenases to all-*trans* RA [80], [81], as observed in livers of males. The increased liver weights (max. increase 65%) probably attenuated the increases in liver all-*trans* RA concentrations in males (max. increase 21%) and exaggerated increases in corresponding amounts (max. increase 93%). Overall, increased all-*trans* RA concentrations may have adverse consequences even if moderately affected, as seen in male livers and female kidneys, since it is involved in the regulation of fundamental processes related to morphogenesis, apoptosis and reproduction via the activation of RAR [80], [81]. The increased retinoid mobilization in livers showed gender differences: the increase in serum retinol of females suggests the release of hepatic retinol into circulation instead of oxidation to all-*trans* RA as seen in males. In addition, there were also marked gender differences in the endogenous tissue levels of several retinoid forms, most notably in the renal retinyl palmitate levels.

Similarly with our findings mobilization of hepatic retinoid stores and increases in renal retinoid levels has been previously reported after treatment with NDL-PCBs 128 [7] and 153 [6], and mono-ortho PCB 105 [62], but also DL-PCBs 126 [63] and 77 [82] as well as TCDD [83]. The dose-dependent and profound decrease of 9-*cis*-4-oxo-13,14-dihydro-RA in livers of female rats (CED 12 mg/kg bw, 55.5 µg/g lipid) is noteworthy as this RA-metabolite binds and activates retinoid receptors and regulates gene transcription both *in vitro* and *in vivo* [84].

It is noteworthy that alterations in retinoid levels took place largely at similar dose levels with decreased thyroid hormone levels (Table 2). These two phenomena have been reported to coexist also after exposure to NDL-PCBs 153 [6] and 128 [7], Aroclor 1254 [85], [86], and TCDD [83], [87], [88]. Thus both AHR dependent and independent mechanisms seem to be involved. Although the possible connection between NDL-PCB –induced alterations of the thyroid and retinoid systems is not known, interactions among RXR, RAR, PXR; CAR, TR and AHR have been reported [89]. In fact, RXRs, activated by retinoid ligands are involved in thyroid hormone transcriptional activity via the thyroid hormone receptor (TR)/RXR heterodimer [89]. Similarly, transcriptionally active forms of PXR and CAR are PXR/RXR and CAR/RXR heterodimers, respectively. Furthermore, retinol binding protein that is responsible for retinol transport in circulation, forms a complex with TTR to avoid glomerular filtration [80], [81]. Therefore the high affinity of hydroxyl-PCBs to TTR may potentially interfere with retinol transport and clearance.

### Liver p53 and DNA damage markers

The study showed that the expression of the tumor suppressor protein p53 and the DNA-damage signaling proteins p53 Ser15, γH2AX Ser139 and pChk2 Thr68 were increased by PCB180 in livers of females. Expression of pMdm2 Ser166 was not affected. This is in line with our recent findings in the human hepatocellular carcinoma cell line HepG2 [90]. In contrast to PCB 180, 13 out of 20 tested NDL-PCBs induced phosphorylation of Mdm2 at Ser166 resulting in attenuated p53 response and lowered basal levels of p53. It is likely that the hydroxyl metabolite of PCB 180 is responsible for the DNA damaging effect. We showed recently that 3′-OH-PCB 180, but not the parent compound induced the expression of DNA damage markers pChk1Ser317 and γH2AXSer319 in HepG2 cells (Al-Anati *et al.*, in preparation).

### Bone effects

In contrast to previous findings with TCDD [91], [92], treatment of young adult rats with PCB 180 resulted only in minor effects on bone. The only dose-dependent effects on bone geometry were decreased cortical area of diaphysis in males and increased trabecular area of metaphysis in females. The latter effect was in contrast to reduced trabecular area observed after treatment with TCDD [92]. Biomechanical testing revealed slightly reduced mechanical strength in terms of decreased yield force of tibial shaft. NDL-PCB 153 was previously shown to alter bone geometry and composition in perinatally exposed goats [93] and in *in utero* exposed sheep fetuses [94]. Thus, bones are potentially sensitive to NDL-PCB, and the low potency and minor severity of the effects in the present study may be due to short duration of exposure as compared to the bone remodellation cycle of about 30 days in rats.

### Gender aspects and sensitivity of different endpoints

**Figure 10:**
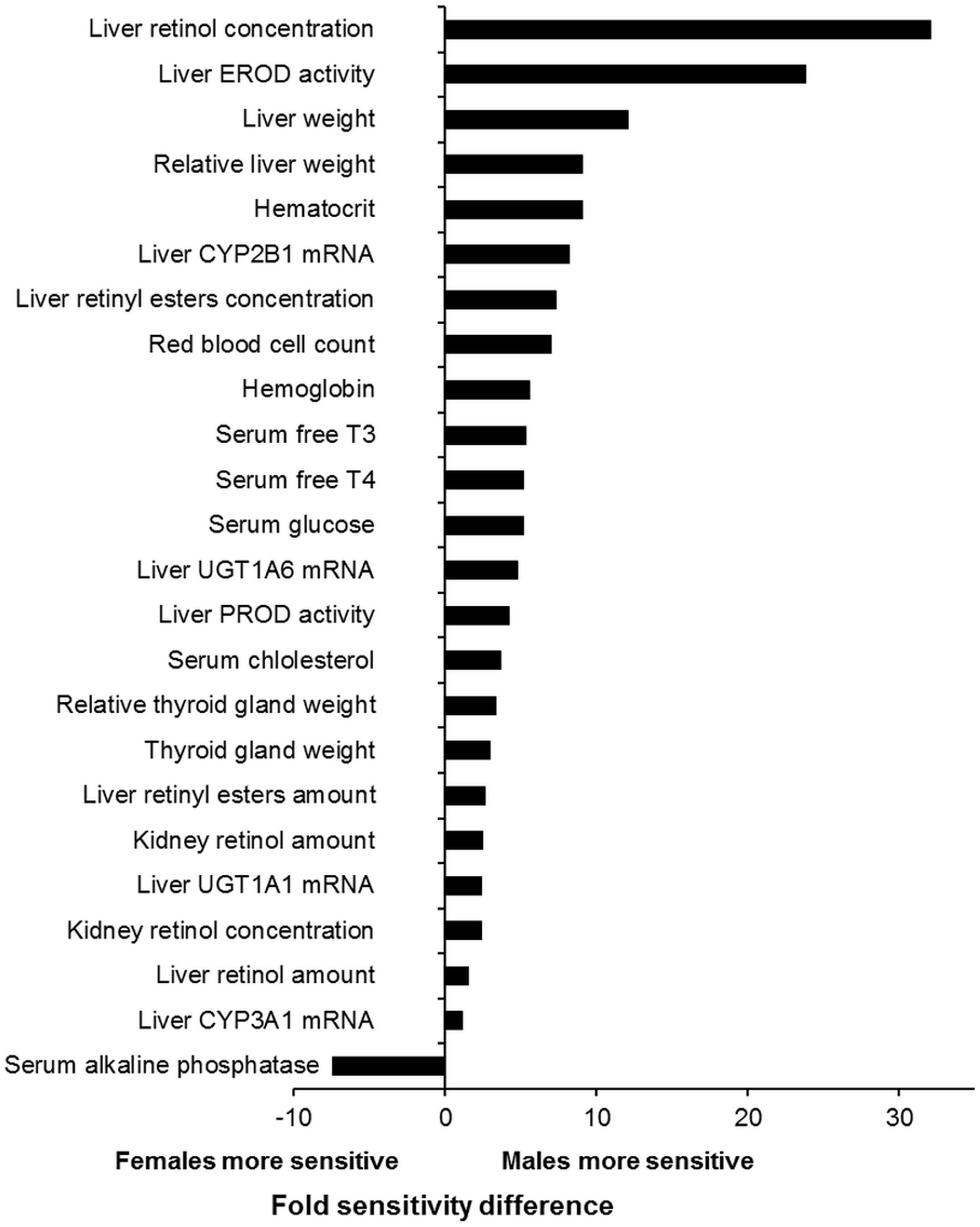
Sensitivity differences between males and females for endpoints showing significant dose-responses. Fold sensitivity difference is shown as the ratio of adipose tissue PCB 180 concentration based CED values at CES 5%.

**Figure 11:**
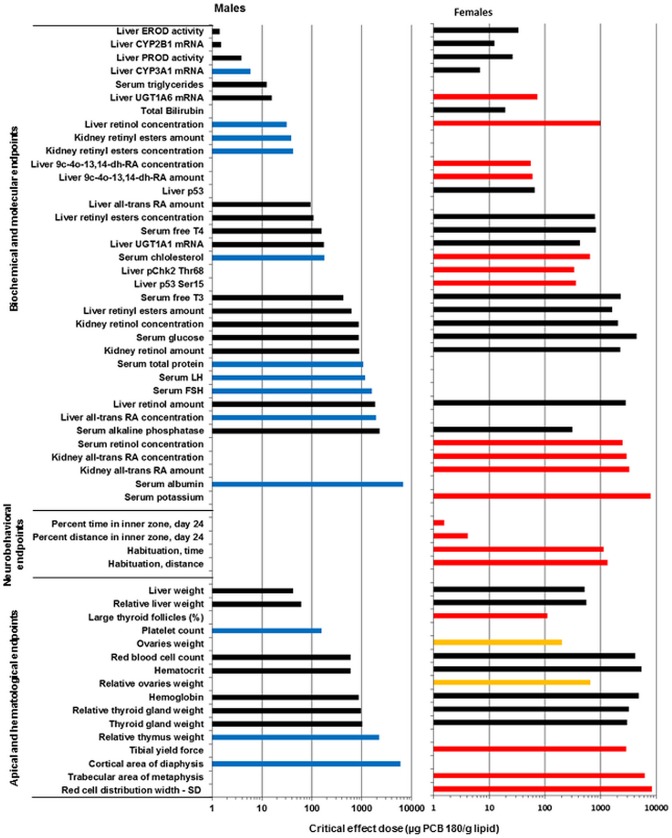
Sensitivity of endpoints showing significant dose-responses in males (left panel) and females (right panel). Adipose tissue PCB 180 concentration based CED values at CES 5% (shown in log scale) are ranked according to sensitivity in males. Endpoints are grouped into biochemical/molecular endpoints, neurobehavioral endpoints and apical/hematological endpoints. Key: endpoints with significant dose-responses in both genders: black bars; endpoints significant only in males: blue bars; endpoints significant only in females: red bars; endpoints existing only in one gender: yellow.

Dose-response modeling with the BMD approach makes it possible to accurately compare gender differences in sensitivity and sensitivity differences of the studied endpoints. Marked gender differences in sensitivity were identified for several endpoints of PCB 180 toxicity (Figs. 10 and 11) indicating different toxicity profiles in males and females.

Males were more sensitive to all endpoints with significant dose-responses in both genders except decreased serum ALP. They included most liver [17] and thyroid related endpoints of which the induction of CYP2B1 mRNA and the associated PROD activity were the most sensitive. The likely explanation is that the higher amount of CAR in cytoplasm of male rat hepatocytes results in sex-dependent induction of *CYP2B1* gene and potentially leads to sexually dimorphic pattern of also other CAR-mediated responses [95], including UGT induction. The fact that a very similar gender difference was observed for the decrease in serum T4 suggests that the more sensitive and more pronounced UGT induction in males is behind the higher sensitivity of males to hypothyroidism. In addition, males were more sensitive to anemia, altered levels of several retinoid parameters, decreased serum glucose and increased serum cholesterol levels. Furthermore, only males showed decreased serum gonadotropin and triglyceride levels, increased serum albumin levels as well as increased liver all-*trans* RA and kidney retinyl ester concentrations and amounts.

It is important to note that in spite of sensitivity of males to majority of the analyzed endpoints only females exhibited altered open field behavior. Nevertheless, this type of altered behavior seems not to be limited to females, because PCB exposure has been earlier reported to result in altered locomotor activity in both genders [48]. This sensitive endpoint (CED-L 0.525 µg/g lipid) was selected for the critical effect for risk characterization (for motivation, see *Risk characterization*). Induction of DNA damage markers was also observed only in females. Accordingly, physiological concentration (10 nM) of 17-β estradiol was shown to amplify 3′-OH-PCB 180 –induced DNA damage in HepG2cells (Al-Anati *et al.*, in preparation). Decreased bone strength, decreased area of large thyroid follicles, hypertrophy of thyroid follicular cells as well as decreased retinoid metabolite 9c-4o-13,14-dh-RA in liver and increased all-*trans*- RA in kidneys were also observed only in females, and females were more sensitive to activation of cells of adrenal cortex *zona fasciculata* (297-fold difference in CED values from semi-quantitative evaluation; Fig. 8). It is therefore obvious that there are not only sensitivity differences among genders, but the toxicity profile of PCB 180 is different in males and females.

Sensitivity ranking of the adipose tissue PCB 180 concentration based CED values for different endpoints is shown in Fig. 11. In males, induction of certain xenobiotic metabolizing enzymes in liver was the most sensitive set of endpoints (CEDs within the range 1–10 µg/g lipid) whereas in females it was the altered locomotor activity. Interestingly these two sets of endpoints were observed at very similar exposure level, and in females the induction of CYP3A1 was almost as sensitive as the percentage of time in inner zone. These observations indicate that both genders are responsive to low doses of PCB180, while the functions affected are partly different. Endpoints with CED values within 10–100 µg/g lipid (in males) included altered retinoid parameters and increased liver weight whereas other endpoints within broad endpoint categories “thyroid effects”, “clinical chemistry” and “retinoid metabolism” showed rather high variability in both genders (CEDs >100 µg/g lipid), and sensitivity of biochemical/molecular endpoints at CED >10 µg/g lipid did not differ from that of apical/hematological endpoints in this regards. On the other hand, all endpoints within “hematology” and “bone effects” showed low sensitivity in both genders.

### Risk characterization

Risk characterization was carried out by using MoE values for the most sensitive endpoints of toxicity and human median exposure values from different cohorts as shown in Table 4. Altered spontaneous locomotor activity has the lowest CED-L value with a clear dose-response and was therefore selected for the critical effect of PCB 180 in young adult rats. Increased serum TSH in males (CED-L 0.04 µg/g lipid) was considered less valid because of high variability (CED/CED-L ratio 6.50). Although species differences in behavior and lack of mechanistic data make it difficult to assess the clinical significance of altered locomotor activity, a variety of PCBs and PCDD/Fs have been shown to induce behavioral effects in animal models at low exposure levels and possibly with different modes-of-action. Because humans are exposed simultaneously to all these compounds, our data emphasize the potential significance of behavioral effects. As pointed out above, altered locomotor activity is a highly integrative behavior subject to modulations by a variety of chemicals and mechanisms, and frequently reported after exposure to PCB congeners and mixtures in different species (reviewed in [48]), including humans [51]. It can therefore be regarded as a potentially relevant endpoint for human health risk assessment. For comparison, CED-L values for induction of several xenobiotic metabolizing enzymes [17] (Table 3, Fig. 11) are quite similar with that of altered locomotor activity indicating that different unrelated targets are affected at low exposure levels of PCB 180. Risk characterization based on altered xenobiotic metabolism would therefore lead to similar outcome.

**Table 4:** Margins of exposure (MoE  =  CED-L/human median PCB 180 concentration) for different endpoints of PCB 180 toxicity and different human cohorts. MoE values exceeding the WHO default uncertainty factor of 25 are bolded.

Endpoint	Gender	CED-L (ng/g lipid)	Human cohort					
			Placenta (Denmark)^1^	Placenta (Finland)^1^	Mother's milk^2^	General population (Finland)^3^	Baltic Sea fishermen^4^	Worst case: the most exposed fisherman^5^
Spontanous locomotor activity	F	525	54.4	94.4	**11.5**	**5.53**	**1.14**	**0.438**
Serum triglycerids	M	7260	752	1306	159	76.5	**15.8**	**6.05**
Liver retinol	M	18800	1948	3381	410	198	40.9	**15.7**
Liver p53	F	30000	3109	5396	655	316	65.2	25
Serum chlolesterol	M	91400	9472	16439	1996	963	199	76.2
Serum thyroxine	M	117000	12124	21043	2555	1233	254	97.5

Human PCB 180 tissue concentration data: median, range (ng/g lipid).

1 Placenta [40] (1997–2001), Denmark: 9.65, 2.03–25.2; Finland: 5.56, 1.44–14.92.

2 Mother's milk, 58 milk pools from 18 European countries [1], [2] (2001–2002): 45.8, 6.1–337.

3 Adipose tissue, general population, Finland [96] (1997–1999): 94.9, 11.3–833.

4 Adipose tissue, Baltic Sea fishermen [45] (1997–1999): 460, 190–1200.

5 Adipose tissue, Baltic Sea fisherman with highest exposure [45] (1997–1999): 120.

MoE values in Table 4 indicate that when using the WHO default UF of 25 and altered locomotor activity as the critical endpoint tolerable PCB 180 tissue concentration is exceeded in human cohorts except the Danish–Finnish joint prospective cohort. Unlike the altered spontaneous locomotor activity (and induction of xenobiotic metabolizing enzymes in liver [17]) all other endpoints of toxicity had clearly higher CED-L values (Table 4, Fig. 11). Tolerable PCB 180 tissue concentration is still exceeded in the Baltic fisherman cohort for decreased serum triglycerides and in the highest exposed fisherman for decreased liver retinol levels. For all other endpoints the MoE is ≥25 for all human cohorts.

## Conclusions

It can be concluded that PCB 180 has a distinct toxicological profile with altered open field behavior in female rats being the most sensitive endpoint, and induction of certain xenobiotic metabolizing enzymes takes place at the same exposure levels in both genders. The profile is partly different in males and females.

Several interacting signaling pathways and nuclear receptor families are involved in mediating the toxic effects of PCB 180. Activation of CAR and PXR lead to the characteristic induction of xenobiotic metabolism, including UGT induction and the subsequent decrease in circulating levels of thyroid hormones. Displacement of thyroid hormones from TTR by hydroxyl metabolites of PCB 180 further contributes to hypothyroidism. Complex nuclear receptor interactions among TRs, CAR, PXR and the retinoid receptors RXR and RAR are likely to play a role in increased mobilization of retinoids and the formation of biologically active retinoid forms. There is also evidence of increased expression of DNA-damage signaling proteins and the tumor suppression protein p53. Furthermore, PCB 180 has some potency to antagonize both androgen and estrogen receptors as well as to inhibit gap junctional intercellular communication *in vitro* [9].

PCB 180 shares several toxicological targets with DL compounds, including changes in behavior, serum lipids, tissue retinoid levels, thyroid gland pathology, thyroid hormone levels and effects on the hematopoietic system. However, the potency of PCB 180 is lower and most likely the mode-of-action different from DL compounds. In addition, PCB180 does not induce several of the characteristic AHR dependent responses, such as thymus atrophy, permanent body weight reduction, and the typical CYP induction profile. This study provides new knowledge for improved PCB risk assessment.

## Supporting Information

Table S1 **Adipose tissue and liver PCB 180 concentrations.**
(XLSX)

Table S2 **Hematology.**
(XLSX)

Table S3 **Clinical chemistry.**
(XLSX)

Table S4 **Thyroid hormones.**
(XLSX)

Table S5 **Steroids and gonadotropins.**
(XLSX)

Table S6 **Tissue retinoid concentrations.**
(XLSX)

Table S7 **Liver and kidney retinoid amounts.**
(XLSX)

Table S8 **Organ weights.**
(XLSX)

Table S9 **Cauda epididymal sperm counts.**
(XLSX)

Table S10 **Tibial geometry, densitometry and biomechanics.**
(XLSX)

Table S11 **Brain aminoacids.**
(XLSX)

Table S12 **Brain dopanine and nicotinic receptor binding.**
(XLSX)

Table S13 **Transthyretin binding of monohydroxy metabolites of PCB 180.**
(XLSX)
